# Systematic Mendelian randomization framework elucidates hundreds of CpG sites which may mediate the influence of genetic variants on disease

**DOI:** 10.1093/hmg/ddy210

**Published:** 2018-06-08

**Authors:** Tom G Richardson, Philip C Haycock, Jie Zheng, Nicholas J Timpson, Tom R Gaunt, George Davey Smith, Caroline L Relton, Gibran Hemani

**Affiliations:** MRC Integrative Epidemiology Unit (IEU), Bristol Medical School (Population Health Sciences), University of Bristol, Oakfield House, Oakfield Grove, Bristol, UK

## Abstract

We have undertaken a systematic Mendelian randomization (MR) study using methylation quantitative trait loci (meQTL) as genetic instruments to assess the relationship between genetic variation, DNA methylation and 139 complex traits. Using two-sample MR, we identified 1148 associations across 61 traits where genetic variants were associated with both proximal DNA methylation (i.e. *cis*-meQTL) and complex trait variation (*P* < 1.39 × 10^−08^). Joint likelihood mapping provided evidence that the genetic variant which influenced DNA methylation levels for 348 of these associations across 47 traits was also responsible for variation in complex traits. These associations showed a high rate of replication in the BIOS QTL and UK Biobank datasets for 14 selected traits, as 101 of the attempted 128 associations survived multiple testing corrections (*P* < 3.91 × 10^−04^). Integrating expression quantitative trait loci (eQTL) data suggested that genetic variants responsible for 306 of the 348 refined meQTL associations also influence gene expression, which indicates a coordinated system of effects that are consistent with causality. CpG sites were enriched for histone mark peaks in tissue types relevant to their associated trait and implicated genes were enriched across relevant biological pathways. Though we are unable to distinguish mediation from horizontal pleiotropy in these analyses, our findings should prove valuable in prioritizing candidate loci where DNA methylation may influence traits and help develop mechanistic insight into the aetiology of complex disease.

## Introduction

The majority of genetic variants associated with complex traits are located in non-coding regions of the genome and therefore likely to influence disease via gene regulation (1). To improve our understanding of these mechanisms, information about genetic variants associated with gene expression (also known as expression quantitative trait loci, eQTL) is now commonly incorporated with complex traits and diseases (2–4). Recently, this type of methodology has been extended to integrate epigenetic data using genetic variants associated with DNA methylation levels (known as methylation quantitative trait loci, meQTL) (5,6). In this study, we have built on previous work to prioritize CpG sites which may play a mediatory role along the causal pathway from genetic variation to complex trait and disease susceptibility.

As with complex traits, DNA methylation levels at CpG sites across the genome can be determined by both genetic and environmental factors. Moreover, observational associations between complex traits and DNA methylation are prone to confounding and reverse causation, which can undermine our ability to infer causal relationships (7,8). An approach to address this limitation is Mendelian randomization (MR), a method by which the causal inference of one trait (the exposure) on another trait (the outcome) can be inferred. This is achieved by using genetic variants known to robustly associate with the exposure as instrumental variables (9,10). The sample size of studies with data on epigenome-wide DNA methylation, genome-wide genetic data and complex traits are modest compared with most genetic association studies of complex traits, primarily due to the current costs of DNA methylation arrays. A recent methodological development to circumvent this limitation is two-sample MR (2SMR), an approach where summary statistics for the effect of genetic instruments on exposure and outcome are obtained from two separate studies (11,12). 2SMR enables causal relationships to be investigated without requiring a sample of individuals with genotype, exposure and outcome data.

**Figure 1.**
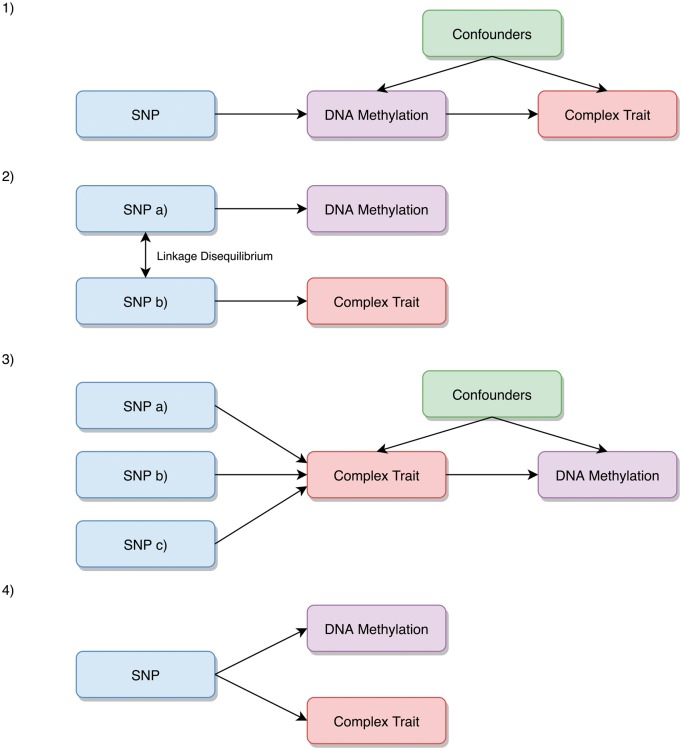
Explanations evaluated which may potentially explain associations between meQTL and trait outcomes. (1) The genetic variant has a causal effect on the complex trait which is mediated by changes in DNA methylation. (2) The genetic variant that influences DNA methylation is in LD with another variant that influences complex trait variation. (3) The genetic variant has a causal effect on the complex trait which subsequently influences DNA methylation at this locus. (4) The genetic variant influences DNA methylation and the complex trait via two independent biological pathways (also known as horizontal pleiotropy).

As described in our previous work (6), when a genetic variant is reliably associated with both DNA methylation and complex trait variation, we postulate that there are four possible scenarios that may account for this (Fig. 1):

1. The genetic variant has a causal effect on the complex trait which is mediated by changes in DNA methylation.
2. The genetic variant responsible for changes in DNA methylation is in linkage disequilibrium (LD) with the genetic variant that influences complex trait variation.
3. The genetic variant has a causal effect on the complex trait (or a related complex trait which resides along the causal pathway to disease) which subsequently influences DNA methylation at this locus.
4. The genetic variant influences DNA methylation and the complex trait via two independent biological pathways (also known as horizontal pleiotropy).

A search for examples where explanation 1 *could* be true is performed by evaluating associations between known meQTLs and complex traits. DNA methylation is typically instrumented by a single *cis*-acting variant, which means that an unreliable MR estimate of causality may arise due to the causal variant for DNA methylation simply being in LD with a causal variant for the complex trait (explanation 2). The chances of this occurrence are dramatically increased when investigating causal relationships systematically as undertaken in our framework. We attempt to distinguish between explanations 1 and 2 using genetic colocalization methods, such as joint likelihood mapping (JLIM), evaluating whether the underlying genetic variation at a genomic region is responsible for effects on both an intermediate and complex trait (13). Genetic colocalization approaches such as JLIM are necessary, but not sufficient, for causality.

 We then attempt to distinguish between explanations 1 and 3 by obtaining instruments for the complex traits and testing the opposite direction of effect (14,15). Using a single *cis*-acting instrument also means that we are unable to reliably distinguish between mediation (explanation 1) and horizontal pleiotropy (explanation 4). Nevertheless, within our framework we use MR to investigate the relationship between DNA methylation and gene expression at loci where mediation is a potential explanation for shared genetic effects. In doing so, we aim to identify a coordinated system of effects through shared genetic variation of molecular phenotypes.

In this study, we have adapted our analytical framework developed previously to map putative causal relationships between DNA methylation and 139 complex traits taken from large-scale consortia using a two-sample framework (16). We build on previous work (5) by extending the survey to a much larger number of traits, interrogating bi-directional relationships, integrating gene expression data into analyses and undertaking exhaustive JLIM analyses to investigate linkage as an explanation for identified effects. Effect estimates on DNA methylation were obtained from the Accessible Resource for Integrated Epigenomics Studies (ARIES) project, which consists of individuals enrolled in the Avon Longitudinal Study of Parents and Children (ALSPAC). Effects on complex traits were obtained using findings from large-scale genome-wide association studies (GWAS). Replication of results with evidence of a putative causal relationship for a selection of traits was undertaken using DNA methylation data from the BIOS QTL browser (17) and complex trait data from up to 334 398 individuals enrolled in the UK Biobank study (18). Functional annotation and enrichment analyses, including data for histone mark peaks and DNAse I hypersensitivity sites across 113 different tissue types, were undertaken for selected variants and CpG sites (19,20).

## Results

### Systematic search for putative mediation of genetic influences on complex traits through DNA methylation

The initial analysis involved over 4.2 million MR analyses to evaluate the potential causal relationship between DNA methylation at 30 328 CpG sites and 139 complex traits using MR-Base (16). We only investigated CpG sites using *cis*-meQTL (i.e. genetic instruments within 1MB distance of their associated CpG site) in order to improve the specificity of the instruments. Subsequently the majority of CpG sites were instrumented using a single *cis*-acting meQTL (*n* = 26 975) and therefore MR effect estimates were calculated using the Wald ratio (21). When more than one instrument was available the inverse variance weighted (IVW) method was used instead (22).

MeQTL effects were typically identified at multiple time points across the life course in ARIES, and therefore we only used effect estimates from a single time point to reduce the burden of multiple testing. A list of the complex traits analysed can be found in Supplementary Material, Table S1, which were selected based on GWAS with effect estimates from over 100 000 genetic variants, sample sizes of over 1000 individuals and undertaken in either European or mixed populations. The MR-Base platform was then used to evaluate the association between our exposure (i.e. DNA methylation levels at a CpG site) and our outcome (i.e. a complex trait). When meQTL effects were obtained from a time point in ARIES which is later in the life course compared with the analysed outcome (e.g. childhood obesity), results should be interpreted as evidence of genetic liability between DNA methylation and complex trait.

There were 1148 putative MR associations between a CpG site and complex trait which survived the multiple testing threshold across 61 different traits (*P* < 1.397 × 10^−08^, Supplementary Material, Table S2). A heat map visualizing the correlation of the *z* scores from the MR analysis across traits can be found in Supplementary Material, Figure S1, which highlights traits which may be influenced by changes in DNA methylation at shared loci.

### Identifying shared genetic variants between DNA methylation and complex traits

Results surviving multiple testing in the previous analysis may arise due to an meQTL and trait-associated variant overlapping at a genomic locus due to chance. To investigate this, we applied the JLIM algorithm (13) which tests whether variation in two traits (i.e. DNA methylation and a complex trait in this study) are driven by a shared causal effect [with the caveat that two causal variants in perfect LD (i.e. *r*^2^ = 1) cannot be investigated using such methodology). This is obtained by generating a permutation-based null distribution for a trait with individual-level data (i.e. DNA methylation in our analysis) and assessing the likelihood that the causal variant for this trait is also responsible for variation on a different trait based on summary-level data (i.e. GWAS results for a complex trait). Permutation testing was implemented by the JLIM method to account for the 1148 associations identified in the previous analysis (*P* < 4.36 × 10^−5^). The JLIM analysis suggested that 348 of the 1148 CpG-trait associations were due to methylation and complex trait variation both being influenced by the same underlying genetic variant (Supplementary Material, Table S3). We refer to these 348 associations hereafter as ‘CpG-trait associations’.

**Figure 2.**
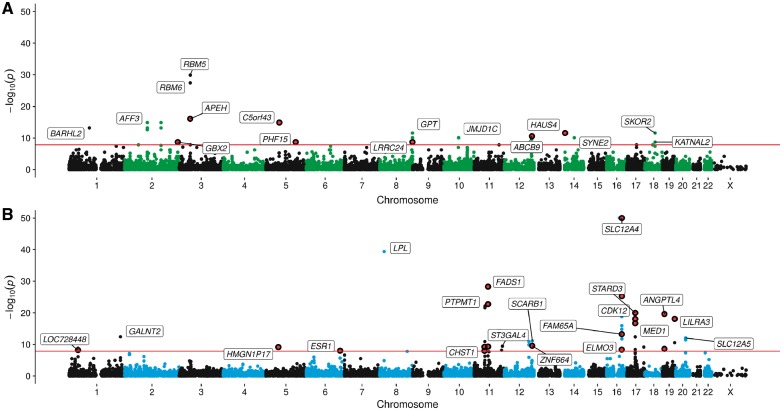
Manhattan plots illustrating results of two-sample MR analysis between epigenome-wide DNA methylation and (**A**) educational attainment (top) and (**B**) HDL cholesterol (bottom). Points represent –log 10 *P*-values (*y*-axis) for CpG sites (genomic location on the *x*-axis) as evaluated using two-sample MR analysis between DNA methylation (as our exposure) and complex traits (as our outcome) using meQTL as genetic instruments. Effects that survive the multiple testing threshold in our analysis (*P* < 1.397 × 10^−08^—represented by the red horizontal line) are annotated using mapped genes according to Illumina (or nearest gene when no gene has been reported by Illumina). Effects where JLIM suggested the causal variant for DNA methylation and complex trait variation were the same are highlighted in red.

Consequently, the 800 associations which did not provide evidence from JLIM in this evaluation were likely due to the causal variant for DNA methylation being in LD with a separate variant responsible for complex trait variation. Figure 2 illustrates findings for 2 of the 61 traits which had at least one effect that survived the multiple testing threshold, where individual points represent *P*-values from the 2SMR analysis. Points highlighted in red correspond to loci where JLIM provided evidence that the same underlying causal variant influences both DNA methylation and complex trait. For example, the results illustrated in Figure 2B suggest that the same causal variants at the *SLC12A4*, *FADS1* and *ANGPTL4* loci are responsible for changes in both proximal DNA methylation and high-density lipoprotein (HDL) cholesterol. In contrast, results suggest the genetic variant driving the observed effect on HDL cholesterol at the *LPL* gene region is not responsible for changes in DNA methylation at this locus. Manhattan plots for all 61 traits can be found in Supplementary File 1.

### Reverse Mendelian randomization

For the 348 CpG-trait associations identified in the previous analysis, we performed reverse MR to test if the CpG-trait associations arose due to traits influencing CpG levels. This was undertaken by modelling a complex trait as our exposure and DNA methylation levels at a CpG as our outcome. The only evidence of association in the reverse MR analysis was between number of cigarettes smoked per day and DNA methylation variation at the *CHRNA5/PSMA4* region (Supplementary Material, Table S4). However, this complex trait currently only has a single genetic instrument which weakens our ability to robustly investigate direction of effect for this result.

The reverse MR analysis was only undertaken for CpG-trait associations detected in the initial analysis due to the anticipated reduction in power when analysing DNA methylation as our outcome. This is highlighted by comparing the average absolute effect estimates obtained from the results of the CpG-trait MR at these CpG sites (beta = 0.216, se = 0.026) against the reverse MR (beta = 0.269, se = 0.263). The lack of associations identified by this analysis is therefore likely due to sample sizes in ARIES, given that similar approaches have identified evidence that complex traits influence DNA methylation using larger samples and methods that require individual level data (23).

### Validation of findings using data from the BIOS QTL browser and UK Biobank study

We undertook a replication analysis for the 128 CpG-trait associations for which independent data was available, repeating analyses using meQTL data from the BIOS QTL browser (17) and complex trait data from the UK Biobank (Supplementary Material, Table S5) (18). There was evidence of replication for 101 of the 128 associations based on multiple testing corrections (*P* < 3.91 × 10^−04^) and the direction of effect between DNA methylation and complex traits (Supplementary Material, Table S6).

### Evaluating the relationship between DNA methylation and gene expression

We integrated gene expression data to investigate whether the genetic variants used to identify CpG-trait associations were known to influence gene expression as well as DNA methylation. Data from the GTEx consortium (24) and the blood eQTL browser (25) suggested that this was the case for 306 of the 348 CpG-trait associations. 2SMR was used to evaluate the relationship between DNA methylation and gene expression at each of these loci, i.e. whether higher DNA methylation associates with higher or lower gene expression (Supplementary Material, Table S7).

These results also provide some biological insight regarding tissue specificity and how this varies for different loci. For example, the effect between *ADIPOQ* gene expression and adiponectin was only observed using adipose subcutaneous data, which is a relevant tissue type for this trait. In contrast, the effect between *ABO* gene expression and various complex traits was found across 24 different tissue types, which can help explain why variation at this region was associated with multiple traits. A summary of the associations detected at each stage of the analysis in the study can be found in Table 1.

**Table 1.** Overview of the main findings from the various stages of this study

Analysis	Objective	Number of tests undertaken	Number of results surviving multiple testing
Two-sample MR (CpG -> complex trait)	Identify potential CpG sites where DNA methylation may mediate the influence of genetic variants on complex traits	4 215 592	1148
Joint likelihood mapping	Assess the likelihood that results from the previous analysis are observed due to two separate causal variants which are in LD with one and other	1148	348
Reverse two-sample MR (complex trait -> CpG)	Evaluate potential evidence for reverse causation, i.e. complex trait influences DNA methylation levels	348	2^a^
Replication two-sample MR	Validate results for 14 complex traits using data from the BIOS QTL browser and UK Biobank study	128	101
Two-sample MR (CpG -> gene expression)	Investigate whether meQTL used as instruments in the initial analysis overlap with variants known to influence nearby gene expression (i.e. whether they are also *cis*-eQTL)	348	306

a Both effects observed in the reverse MR analysis were based on a single genetic instrument, similar to findings in the initial MR at these loci (CpG -> complex trait). We are therefore unable to robustly distinguish the direction of effect between methylation and complex trait for these associations.

### Gene prioritisation, implicated biological pathways and druggable targets

A suite of bioinformatics tools was used to calculate the predicted consequences and severity for genetic variants responsible for CpG-trait associations (Supplementary Material, Table S8). Likely impacted genes for CpG-trait associations were prioritized using DEPICT (26) (Supplementary Material, Table S9).

Annotated genes were grouped into categories based on their associated trait (Supplementary Material, Table S10). Each group of genes was then analysed in turn using ConsensusPathDB (27) to test whether likely implicated genes were enriched for biological pathways (Supplementary Material, Table S11) and gene ontology terms (Supplementary Material, Table S12) based on a false discovery rate < 5%. Overall there were 64 enriched pathway effects and 232 enriched GO term effects. Amongst these enrichments are biologically meaningful results, such as genes associated with cardiovascular traits being enriched for lipid and metabolic processes, whereas genes associated with autoimmune traits are enriched for immune system pathways and terms.

Prioritised genes were also evaluated for druggability using the ChEMBL database (28) (version 23 accessed on 13 June 2017). Proteins encoded by implicated genes which are targets for therapeutic intervention were identified (Supplementary Material, Table S13). These included approved drugs, such as estropipate and estradiol cypionate, which targets *ESR1* (associated with HDL cholesterol and birth weight), as well as compounds in development, such as cyclin-dependent kinase inhibitors, which target *CDK12* (associated with HDL cholesterol and serum creatinine).

### Tissue-specific enrichment for CpG sites

CpG sites implicated in CpG-trait associations were annotated to determine whether they reside in regulatory regions using data from Illumina and Ensembl (29). DNAse I and histone mark peak data across 113 different tissue types from the ENCODE and the Roadmap Epigenomics projects was also used to annotate CpG sites (19,20). CpG sites were then grouped according to the category of their associated trait (Supplementary Material, Table S10) and tested for enrichment after removing proximal probes which may be co-methylated (Supplementary Material, Tables S14–22). In particular, evidence of enrichment for H3K4me1 histone marks was observed for associated CpG sites, as well as evidence of enrichment in tissue types relevant for associated traits. For instance, the top hit for autoimmune traits was observed for H3K4me1 marks in spleen tissue, whereas the top hit for haematological traits was observed for H3K4me1 marks in primary haematopoietic cells. Heat maps illustrating these results for histone mark peaks across different tissue types can be found in Supplementary Material, Figure S2.

## Discussion

In this study we have extended an analytical framework to systematically evaluate the potential causal relationship between DNA methylation and complex traits using GWAS summary data. We identified 348 associations where CpG sites and complex disease share genetic influences. Although we are unable to robustly demonstrate that these effects occur along a common causal pathway to disease (e.g. the associations could be compatible with horizontal pleiotropy), we found that 306 of these associations also share genetic loci which influence gene expression. The genes impacted by changes in DNA methylation at these CpG sites represent promising candidates to explore the potential mediatory role of epigenetic modifications and their potential downstream effects on disease aetiology.

An attractive advantage of using 2SMR to investigate CpG–trait relationships is that it circumvents the requirement of having both intermediate and complex traits measured in the same sample. For instance, a recent epigenome-wide association study (EWAS) of lipids used a sample size of 725 individuals in their discovery analysis to identify two CpG sites associated with HDL cholesterol (30). However, as illustrated in the bottom plot of Figure 2, using findings from a large-scale genetic association study (with approximately 190 000 individuals) we have discovered nine genetic loci (which are different to the two identified in the aforementioned EWAS), which may influence HDL cholesterol variation via changes in DNA methylation. Furthermore, by using genetic instruments we avoid the common pitfalls in observational studies (e.g. EWAS) of confounding and reverse causation. An example of this can be found by contrasting the top plot in Figure 2 with results from a recent EWAS of educational attainment, which identified associations at nine CpG sites that were all previously associated with cigarette smoking (31). Although educational attainment may be an underlying cause of these changes in methylation levels (i.e. educational attainment influences smoking behaviour), such claims cannot be made with confidence in the presence of confounding factors. In contrast, none of the six independent CpG sites linked with educational attainment in this study are associated with exposure to cigarette smoking. This is based on findings from the largest smoking EWAS to date of both own smoking (32) and exposure to maternal smoking *in utero* (33).

The framework used in this article is unlikely to be able to uncover novel trait-associated loci in the field of GWAS because the experiment-wide multiple testing correction is similar to the canonical GWAS significance threshold. But the framework can potentially uncover evidence suggesting that changes in DNA methylation may influence traits at these loci. In terms of specific loci detected in our framework where this may be the case, we have been able to support previously reported findings as well as build upon them. For instance, there is increasing evidence that changes in DNA methylation may influence cardiovascular traits at the *ADCY3*, *ADIPOQ* and *FADS1* loci, which supports results detected by previous studies (5,6). However, by using meQTL data derived from a larger sample of individuals and also GWAS data for a far greater number of complex traits, we have been able to detect novel loci where DNA methylation may play a role in disease susceptibility. For example, previous findings have only implicated CpG sites on chromosome 6, predominantly in the MHC region, with risk of rheumatoid arthritis (34). In our study, there were six novel loci outside of this region where genetic variation may influence rheumatoid arthritis risk via changes in DNA methylation (*TTC34, MMEL1, AFF3, IRF5, CXCR5* and *PGAP3*). Furthermore, our pathway and gene ontology enrichment analyses provide evidence that sets of genes detected in our study may collectively influence disease. For example, the strongest evidence of pathway enrichment for autoimmune-related genes was for the inflammatory bowel disease pathway according to the Kyoto Encyclopaedia of Genes and Genomes database (35). This effect was driven by the *STAT3, IL18R1* and *SMAD3* genes which have previously been implicated in inflammatory bowel disease (36–38).

In this study, we have used DNA methylation derived from blood to investigate its effect on a range of complex traits, although epigenetic processes are known to be tissue specific (39,40). For instance, we have identified association signals for cognitive and neurological traits where we may expect the causal genes to be expressed in brain tissue. However, studies have demonstrated that the correlation between DNA methylation in measures of blood and brain is stronger than can be accounted for by chance (41,42). This supports the validity of the findings presented in this study for traits where blood may not be a relevant tissue, although we suggest that in-depth tissue-specific evaluations are necessary to explore these further. Further evaluations are also warranted to investigate temporal relationships between DNA methylation and complex traits. For instance, in this study the meQTL we used had effect sizes that were typically consistent across the five time points within the ARIES project. We have included all meQTL results for all five time points for the 348 associations identified in this study in Supplementary Material, Table S23 to illustrate this point. We find only two meQTL were associated at only a single time point. This could help facilitate future analyses which investigate how early in the life course changes in DNA methylation may occur with respect to disease progression.

The 450K Illumina Infinium Beadchip array used to generate the DNA methylation data in this study only covers ∼1.7% of the 29 million CpG sites across the human genome. This suggests that a wealth of unmeasured data remains unexplored within this paradigm. Furthermore, although we have demonstrated the value of our analytical framework to investigate the role of DNA methylation in disease, we anticipate future studies will have success by investigating other intermediate traits in a similar manner, such as histone marks, metabolites and proteins. These endeavours will be valuable in uncovering signals which reflect a coordinated system of causality, as well as helping pinpoint the true causal gene at densely populated gene neighbourhoods. They should also prove particularly valuable to help identify and evaluate targets for therapeutic intervention.

Studies with increasingly large sample sizes with ‘omic’ data will also allow more robustly associated QTL across different omics types to be uncovered across the genome. This will be hugely beneficial for frameworks such as the one portrayed in this study as it should improve causal inference amongst intermediate traits and downstream implications on disease susceptibility. Moreover, using multiple instruments can improve our ability to separate mediation from horizontal pleiotropy as the putative mechanism underlying the association (43–45). The integration of colocalization methods to assess whether changes in DNA methylation and complex traits are driven by shared causal variants will remain important to implement. In this study, we have been able to use the JLIM method due to having individual level data on epigenome-wide DNA methylation from the ARIES project. Future endeavours, which may be restricted to using summary-level data for omics trait, are able to utilize viable alternatives, such as the HEIDI (heterogeneity in dependent instruments) (2) and ‘coloc’ (46) methods. An illustration of the importance of such approaches can be found when evaluating the results at the *ABO* locus in our study. In the initial MR analysis, there were association signals with seven different traits at this locus, although after applying the JLIM method only detected associations with haemoglobin concentration, red blood cell count and myocardial infarction remained.

A limitation of using 2SMR is that the statistical power is determined by the sample size used to generate effect estimates on the outcome variable. In this study, we therefore only applied the reverse MR analysis at loci identified in the initial analysis (i.e. loci where a SNP already exhibits a large enough effect on a complex trait for it to be an meQTL). Nonetheless, we did not identify strong evidence that complex traits influence DNA methylation levels in our reverse MR analysis, although this approach is likely to yield insightful findings as larger samples with 450K data become accessible. However, subsequent studies which investigate this need to take into complex trait incidence. For example, in the ARIES cohort it is unlikely that incidence of coronary heart disease would have been frequent enough to identify a true causal effect on DNA methylation regardless of sample size. Therefore results can only be regarded as an association of the disease/trait liability as opposed to causality. Furthermore, effect estimates for the instrumental variables used in our study were obtained from the same sample that they were identified in. Future studies which have access to meQTL data from multiple cohorts should also benefit from identifying instruments in a separate dataset to those which effect estimates are derived from to reduce the influence of winner’s curse on the MR effect estimates.

The results presented in this study are likely only the tip of the iceberg for candidate loci which may influence complex traits via epigenetic mechanisms. Thorough evaluations of these loci are necessary to determine the extent to which these processes play a role in complex disease risk. A wealth of data on intermediate omic traits are expected to be generated in large sample sizes across multiple tissue types in forthcoming years. MR can be used to evaluate relationships between these intermediate traits and help develop our understanding of the putative causal pathway from genetic variation to disease.

## Materials and Methods

### Overview

In this study we attempt to create a mapping of SNPs known to influence DNA methylation levels that also influence complex traits. Such a relationship is necessary (but not sufficient) for DNA methylation to lie on the causal path from genetic polymorphisms to complex traits. We build on a previously described analytical strategy by expanding the analysis to a large number of complex traits and diseases. Briefly, we use previously published results from meQTL studies to test 30 328 CpG sites with known genetic factors (the ARIES dataset, a subset of participants from ALSPAC). Each CpG site is tested for association with each of 139 complex traits, using published GWAS summary data that were compiled in the MR-Base database. Putative findings are replicated using meQTL results from the BIOS dataset and genetic associations with complex traits from UK Biobank.

### The Avon Longitudinal Study of Parents and Children (ALSPAC)

ALSPAC is a population-based cohort study investigating genetic and environmental factors that affect the health and development of children. The study methods are described in detail elsewhere (47,48) (http://www.bristol.ac.uk/alspac). Briefly, 14 541 pregnant women residents in the former region of Avon, UK, with an expected delivery date between 1 April 1991 and 31 December 1992, were eligible to take part in ALSPAC. Detailed information and biosamples have been collected on these women and their offspring at regular intervals, which are available through a searchable data dictionary (http://www.bris.ac.uk/alspac/researchers/data-access/data-dictionary/).

Written informed consent was obtained for all study participants. Ethical approval for the study was obtained from the ALSPAC Ethics and Law Committee and the Local Research Ethics Committees.

### Accessible Resource for Integrated Epigenomic Studies (ARIES) project

#### Samples

The ARIES study is a subset of the participants in the ALSPAC study. Blood samples were obtained for 1018 mother–offspring pairs (mothers at two time points and their offspring at three time points) (49). The Illumina HumanMethylation450 (450K) BeadChip array was used to measure DNA methylation at over 480 000 sites across the epigenome.

#### Methylation assays

DNA samples were bisulfite treated using the Zymo EZ DNA Methylation^TM^ kit (Zymo, Irvine, CA). The Illumina HumanMethylation450 BeadChip (HM450k) was used to measure methylation across the genome and the following arrays were scanned using Illumina iScan, along with an initial quality review using GenomeStudio. A purpose-built laboratory information management system (LIMS) was responsible for generating batch variables during data generation. LIMS also reported quality control (QC) metrics for the standard probes on the HM450k for all samples and excluded those which failed QC. Data points with a read count of 0 or with low signal: noise ratio (based on a *P*-value > 0.01) were also excluded based on the QC report from Illumina to maintain the integrity of probe measurements. Methylation measurements were then compared across time points for the same individual and with SNP-chip data (HM450k probes clustered using *K*-means) to identify and remove sample mismatches. All remaining data from probes was normalized with the Touleimat and Tost (50) algorithms using R with the wateRmelon package (51). This was followed by rank-normalizing the data to remove outliers. Potential batch effect were removed by regressing data points on all covariates. These included the bisulfite-converted DNA (BCD) plate batch and white blood cell count which was adjusted for using the *estimateCellCounts* function in the minfi Bioconductor package (52).

#### Genotyping assays

Genotype data were available for all ALSPAC individuals enrolled in the ARIES project, which had previously undergone QC, cleaning and imputation at the cohort level. ALSPAC offspring selected for this project had previously been genotyped using the Illumina HumanHap550 quad genome-wide SNP genotyping platform (Illumina, Inc., San Diego, CA, USA) by the Wellcome Trust Sanger Institute (WTSI, Cambridge, UK) and the Laboratory Corporation of America (LCA, Burlington, NC, USA). Samples were excluded based on incorrect sex assignment; abnormal heterozygosity (<0.320 or >0.345 for WTSI data; <0.310 or >0.330 for LCA data); high missingness (>3%); cryptic relatedness (>10% identity by descent) and non-European ancestry (detected by multidimensional scaling analysis). After QC, 500 527 SNP loci were available for the directly genotyped dataset. Following QC the final directly genotyped dataset contained 526 688 SNP loci.

#### Imputation

Genotypes with MAF > 0.01 and Hardy-Weinberg equilibrium *P* > 5 × 10^−7^ were phased together using ShapeIt (version 2, revision 727) (53) and imputed using the 1000 Genomes reference panel (phase 1, version 3, phased using ShapeIt version 2, December 2013, using all populations) using Impute (v2.2.2) (54). After imputation dosages were converted to bestguess genotypes and filtered to only keep variants with an imputation quality score ≥ 0.8. The final imputed dataset used for the analyses presented here contained 8 074 398 loci.

### The meQTL database

Effects for genetic variants known to strongly associate with DNA methylation (referred to hereafter as meQTL), as estimated using the ARIES dataset in a previous study, were obtained from the meQTL database (http://www.mqtldb.org/; date last accessed September 9, 2017) (55). In this study we have only used meQTL acting in *cis* (i.e. variants located within 1MB of their associated CpG site) to reduce the risk of pleiotropy influencing our results, as variants which are associated with methylation levels at multiple loci across the genome may be more likely to impact independent biological pathways simultaneously.

LD clumping was undertaken to identify independent meQTL (*r*^2^ < 0.01) for each CpG site which could be used as instrumental variables for MR analyses based on an inclusion criteria of *P* < 1.0 × 10^−7^. Only *cis*-meQTL were evaluated (i.e. SNPs associated with DNA methylation at a CpG site within a 1MB distance) as trans-meQTL may be more prone to horizontal pleiotropy. Based on this, there were 30 328 CpG sites eligible for analysis (26 975 CpG sites with 1 meQTL, 5984 CpG sites with 2 meQTLs, 969 CpG sites with 3 meQTLs, 140 CpG sites with 4 meQTLs and 3 CpG sites with 5 meQTLs). As effect estimates for meQTL were typically consistent across time points in ARIES, we only ran analyses once for each CpG site to reduce the burden of multiple testing. This should therefore facilitate future analyses for studies with specific hypotheses regarding temporal changes in DNA methylation. Supplementary Material, Table S23 provides effects across all time points for SNP–CpG combinations which were identified by our study. An overview of the number of *cis*-meQTLs at each time point in ARIES along with descriptive summary statistics can be found in Supplementary Material, Table S24.

### GWAS summary data for 139 complex traits and diseases

We extracted effects of genetic variants on complex traits using large-scale studies which were available within the MR-Base platform (http://www.mrbase.org; date last accessed September 9, 2017) (16). We used the following inclusion criteria to select complex traits to be analysed:

- Effects reported genome-wide for over 100 000 genetic variants
- Study samples must be larger than 1000
- Either European or mixed populations
- Reported beta, standard error and effect alleles for variants

These criteria yielded 139 complex traits and diseases (Supplementary Material, Table S1).

### The BIOS QTL browser

The BIOS QTL browser contains results from meQTL analyses in whole blood using a sample of 3841 Dutch individuals (http://www.genenetwork.nl/biosqtlbrowser/; date last accessed September 9, 2017) (17). The full list of primary *cis*-meQTLs was downloaded to evaluate effects identified in the discovery MR analysis conducted in ARIES.

### The UK Biobank

Genotype data was available for approximately 490 000 individuals enrolled in the UK Biobank study. Phasing and imputation of this data is explained elsewhere (56). Individuals with withdrawn consent, evidence of genetic relatedness or who were not of ‘white European ancestry’ based on a *K*-means clustering (*K* = 4) were excluded from analysis.

Phenotype data were collected for the following traits (with their UK Biobank variable ID in brackets) which were identified as suitable for replication due to their samples sizes after merging with genotype data (*n* > 1000); age at menarche (2714), age at menopause (3581), asthma (22 127), birth weight (20 022), body mass index (21 001), cigarettes smoked per day (3456), extreme height (derived from 50), height (50), hip circumference (49), myocardial infarction (41 202, ICD10 code = I21 or I22), obesity class 1 (derived from 21 001), type 2 diabetes (derived from 2443, although this variable does not distinguish between diabetes type), waist circumference (48), weight (21 002) and years of schooling [derived from 6138 to calculate EduYears as described by Okbay *et al.* (57)]. After exclusions there were up to 334 398 individuals with both genotype and phenotype data who were eligible for analysis.

### The GTEx consortium and blood eQTL browser

Tissue-specific eQTL data was downloaded the GTEx portal (https://gtexportal.org/; date last accessed September 9, 2017) (version v6p). When effect estimates for meQTL were not available from GTEx we obtained estimates for a surrogate variants (*r*^2^≥ 0.8). Finally, when there was no surrogate variants available we consulted the blood eQTL browser (https://genenetwork.nl/bloodeqtlbrowser/; date last accessed September 9, 2017) (25).

### Statistical analysis

#### Identifying candidate loci for mediation by DNA methylation

2SMR was undertaken systematically to evaluate evidence of a causal relationship between DNA methylation at all eligible CpG sites and complex traits. In this initial analysis DNA methylation was treated as our exposure and complex traits as our outcome, using meQTL as our instrumental variables. We used the PhenoSpD method (58–60) to calculate the appropriate number of independent traits to adjust our analysis for due to strong correlation amongst certain traits (i.e. BMI and obesity). The multiple testing threshold was calculated as 0.05 divided by the derived number of independent tests. CpG sites for effects which survived this threshold were annotated based on evaluations of the 450K array (61,62). When only one genetic instrument was available MR effect estimates are based on the Wald ratio test (21):

$${\hat{\beta }}_{Wald\;ratio}=\;\frac{{\hat{\beta }}_{Y|Z}\;}{{\hat{\beta }}_{X|Z}}$$

$${se(\hat{\beta }}_{Wald\;ratio})\;=\sqrt{\frac{se({{\hat{\beta }}_{Y|Z})\;}^{2}}{{{\hat{\beta }}_{X|Z}}^{2}}+\;\frac{{{\hat{\beta }}_{Y|Z}}^{2}se({{\hat{\beta }}_{X|Z})\;}^{2}}{{{\hat{\beta }}_{X|Z}}^{4}}-\;\frac{2{\hat{\beta }}_{Y|Z}cov({\hat{\beta }}_{X|Z},{\hat{\beta }}_{Y|Z})\;}{{{\hat{\beta }}_{X|Z}}^{3}}\;}$$

where $\hat{\beta }\;Y|Z$ is the coefficient of the genetic variant in the regression of the exposure (e.g. DNA methylation) and $\hat{\beta }\;X|Z$ is the coefficient of the genetic variant in the regression of the outcome (e.g. complex trait).

Where two or more genetic instruments were available for analysis we used the IVW method to obtain MR effect estimates (22):

$${\hat{\beta }}_{IVW}=\;\frac{\sum_{k}X_{k}\;Y_{k}\;\sigma_{Yk}^{-2}}{\sum_{k}X_{k}^{2}\;\sigma_{Yk}^{-2}}$$

$$se({\hat{\beta }}_{IVW})=\;\sqrt{\frac{1}{\sum_{k}X_{k}^{2}\;\sigma_{Yk}^{-2}}}$$

Where *X* is our exposure, *Y* is our outcome and our genetic variants are *k* (where *k* = 1,…, *n*).

We used the MR-Base database and software to conduct this analysis. We provided the effect size estimates for the genetic effects on CpGs, and then extracted the corresponding SNP effects from the GWAS summary data of the complex trait outcomes. The TwoSampleMR R package was used to interface with the MR-Base database and to perform the IVW and Wald ratio calculations. If meQTL effect estimates on a GWAS trait are not available, MR-Base attempts to find a genetic variant in strong LD (*r*^2^ ≥ 0.8) with the relevant meQTL to act as a proxy. Data harmonization is also undertaken to ensure that effect estimates for SNPs are based on the same strand (i.e. the ‘effect alleles’ for reported findings are the same). Results from 2SMR analyses were illustrated as Manhattan plots using code derived from the qqman package in R (63).

#### Distinguishing causal effects from genetic confounding due to linkage disequilibrium

Results which survived the multiple testing threshold in the previous analyses were evaluated using the joint likelihood method (JLIM) (13). The JLIM method evaluates whether the same underlying genetic variation is responsible for effects on two traits (i.e. DNA methylation at a CpG site and a complex trait in this study). This is achieved using individual-level data for one trait, which was DNA methylation levels obtained from the ARIES project in this study, to generate a permutation-based null distribution. The number of permutations required by the JLIM method was determined by number of tests undertaken (i.e. the number of associations which survived the *P*-value threshold in the previous analysis). A lack of evidence (i.e. *P* < 0.05/number of associations evaluated) in this analysis would suggest that the causal variant for methylation variation was simply in LD with the putative causal variant for the trait (thus introducing genetic confounding into the association between DNA methylation and complex trait).

The JLIM approach was selected over alternative colocalization methods [such as the HEIDI (heterogeneity in dependent instruments) (2) and ‘coloc’ methods (46)] as in this study we always had individual-level data for one of the traits being assessed (epigenome-wide DNA methylation levels from the ARIES project) and therefore did not have to rely on availability of summary statistics for both traits. The authors of the JLIM method also demonstrate strong overall performance compared with alternative approaches, although they do specify two limitations to ensure accurate detection of shared genetic effects between two traits. These limitations are that their resolution becomes limited when (1) at high LD levels (i.e. *r*^2^ ≥ 0.8) between multiple causal instruments and (2) when the QTL effect (i.e. meQTL in this study) is very weak (i.e. *P* > 0.01). These were addressed in our study as we only used multiple instruments within the MR analysis that were independent (*r*^2^ < 0.01) and strongly associated with DNA methylation (*P* < 1.0 × 10^−7^).

#### Reverse Mendelian randomization

For CpG-trait effects identified in the previous analysis, we also used 2SMR to evaluate evidence of genetic liability by modelling complex traits as our exposure and DNA methylation as our outcome. Instruments for complex traits were selected based on a threshold of 5.0 × 10^−08^ from large-scale GWAS after LD clumping to identify independent variants. The IVW method was applied to estimate the causal effects of traits on CpG sites where more than one instrument was available, otherwise the Wald ratio was used.

#### Replication of effects using the BIOS QTL browser and UK Biobank

For CpG-trait associations where DNA methylation and complex trait were driven by the same causal variant, as inferred by the JLIM method, we repeated our initial analysis using meta-analysed meQTL data from the BIOS QTL browser (17) and trait data from the UK Biobank project (18).

This validation analysis was undertaken for associations across 14 traits from the full release of the UK Biobank project for which large sample sizes (*n* ≥ 10 000) were available after merging with available genetic data (Supplemental Material, Table S4) (18). Linear or logistic regression was used (depending on whether the trait was continuous or binary respectively) to determine effect estimates of genetic variants on complex traits adjusted for age, sex, the first 10 principal components and a binary indicator which reflects which genotype chip individuals were measured on. This was because a subset of UK Biobank individuals had their genotype measured on the Affymetrix UK BiLEVE Axiom array (∼50 000 participants), whereas the remainder were measured using the Affymetrix UK Biobank Axiom array.

#### Causal relationship between DNA methylation and gene expression

We undertook 2SMR to evaluate the relationship between DNA methylation and gene expression for effects where the causal variant, as indicated by the JLIM method described above, was both an meQTL and eQTL. Effect estimates for variants on gene expression were obtained from the GTEx consortium v6p (www.gtexportal.org/; date last accessed September 9, 2017) (64). When effect estimates for the putative causal variant were not available from GTEx we identified a surrogate variant instead (*r*^2^≥ 0.8). Where no surrogate was available within GTEx we consulted the blood eQTL browser (http://genenetwork.nl/bloodeqtlbrowser/; date last accessed September 9, 2017) (25).

### Functional informatics

#### Variant annotation and gene prioritization

Genetic variants for effects potentially mediated by changes in DNA methylation were analysed using the variant effect predictor (VEP) (65) to calculate their predicted consequence. Regulatory data were obtained from Ensembl (www.ensembl.org/; date last accessed September 9, 2017) (29) to evaluate whether these variants reside within regulatory regions of the genome.

Prior to enrichment analyses and gene prioritization, as effects were grouped together as opposed to evaluated individually, we removed effects involving CpG sites flagged for exclusion based on evaluations by Zhou *et al.* (62) and Naeem *et al.* (61). This was based on their criteria of overlapping SNPs at CpG probes, probes which map to multiple locations and repeats on the 450K array. These CpGs were not removed at an earlier stage as they may still warrant further evaluations by other studies, although all subsequent analyses in our pipeline require aggregating multiple CpGs together. The DEPICT method (data-driven expression-prioritized integration for complex traits) (26) was used to prioritize genes for all remaining variants. Variants which were not allocated a likely impacted gene by DEPICT were annotated with their nearest gene using bedtools (66).

#### Pathway and gene ontology enrichment

Genes implicated in the previous evaluations were tested for enrichment of functional pathways and gene ontology terms using ConsensusPathDB (27). When multiple genes were implicated at the same association signal we used annotations according to DEPICT over the nearest gene. All results which had a false discovery rate < 5% were reported.

#### Identifying known and candidate genes for therapeutic intervention

We consulted the ChEMBL database (28) (version 23 accessed on 13 June 2017) to ascertain whether any of the implicated genes encode proteins for known targets of approved drugs or compounds in development.

#### Tissue-specific enrichment for CpG sites

The hypergeometric test was used to test for enrichment of implicated CpG sites for histone mark peaks and regions of DNAse I in up to 113 different tissue and cell types from the *Encyclopedia of DNA Elements* (ENCODE) and Roadmap Epigenomics projects. To calibrate background expectations, we randomly selected CpG sites across the epigenome which resided in similar genomic regions based on Illumina annotations (i.e. CpG island, gene body etc.). We used permutations to control for multiple testing by randomly selecting the same number of implicated CpG sites matched on location and then repeating the enrichment computation for 10 000 iterations. This analysis was repeated using regulatory annotations from the Illumina 450K file (enhancer regions) and Ensembl (promoters, open chromatin regions, transcriptional repressor CTCF sites and transcription factor binding sites).

## Supplementary Material

Supplementary Data
