# Supplementary material for: Systematic Mendelian randomization framework elucidates hundreds of CpG sites which may mediate the influence of genetic variants on disease
Source: Hum Mol Genet. 2018 Jun 8;27(18):3293–304. doi: 10.1093/hmg/ddy210 (PMC6121186; doi:10.1093/hmg/ddy210)
Supplement: Supplementary Data [file ddy210_supp_data.zip › Legends to Supplementary Figures.docx]

# Legends to Supplementary Figures

**Supplementary Figure 1: A heat map illustrating the correlation between complex traits based on Mendelian randomization analyses with DNA methylation**

Correlations between 139 complex traits using Z scores derived from Mendelian randomization analyses using DNA methylation as our exposure. Positive correlations are represented in blue, whereas negative correlations are red. The colours of traits labels correspond to their allocated category as described in Supplementary Table 1.

**Supplementary Figure 2: Heat maps representing evidence of enrichment of identified CpGs within histone marks**

These heat maps visualise enrichment p-values as described in Supplementary Tables 14-22. Segments which are dark blue represent the strongest evidence of enrichment that associated CpG sites identified by this study reside in tissue-specific histone marks more than can be accounted for by chance. Histone peak data was obtained from the Roadmap Epigenomics project.
