# Supplementary material for: Systematic Mendelian randomization framework elucidates hundreds of CpG sites which may mediate the influence of genetic variants on disease
Source: Hum Mol Genet. 2018 Jun 8;27(18):3293–304. doi: 10.1093/hmg/ddy210 (PMC6121186; doi:10.1093/hmg/ddy210)
Supplement: Supplementary Data [file ddy210_supp_data.zip › Supplementary File 1.pdf]

Figure legend

These Manhattan plots illustrate results from evaluating the causal influence of DNA methylation on complex traits using genetic variants as instruments. All points correspond to p-values derived from the Mendelian randomization analysis as described in the manuscript. Points which are highlighted mean that these effects provided evidence that the same underlying genetic variation was observed to influence both methylation and trait at this locus using the joint likelihood mapping method. Plots are coloured depending on the subcategory that the analysed traits has been allocated to, as there is more likely to be shared loci across traits within the same category. P-values have been capped at 1×10^{-50} to ensure plots remain legible.

Aging

Age at menarche

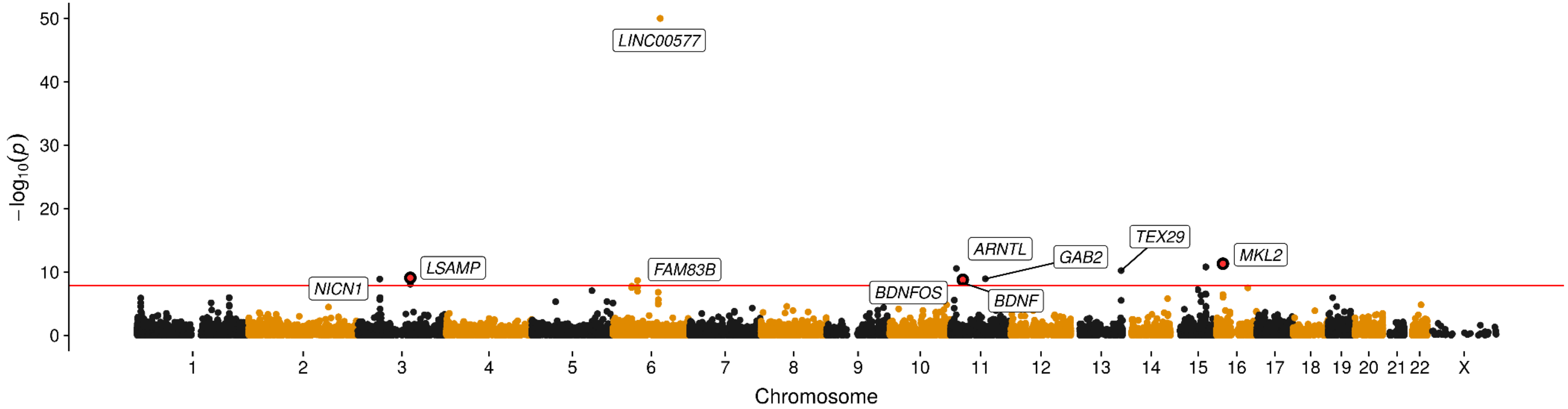

Age at menopause

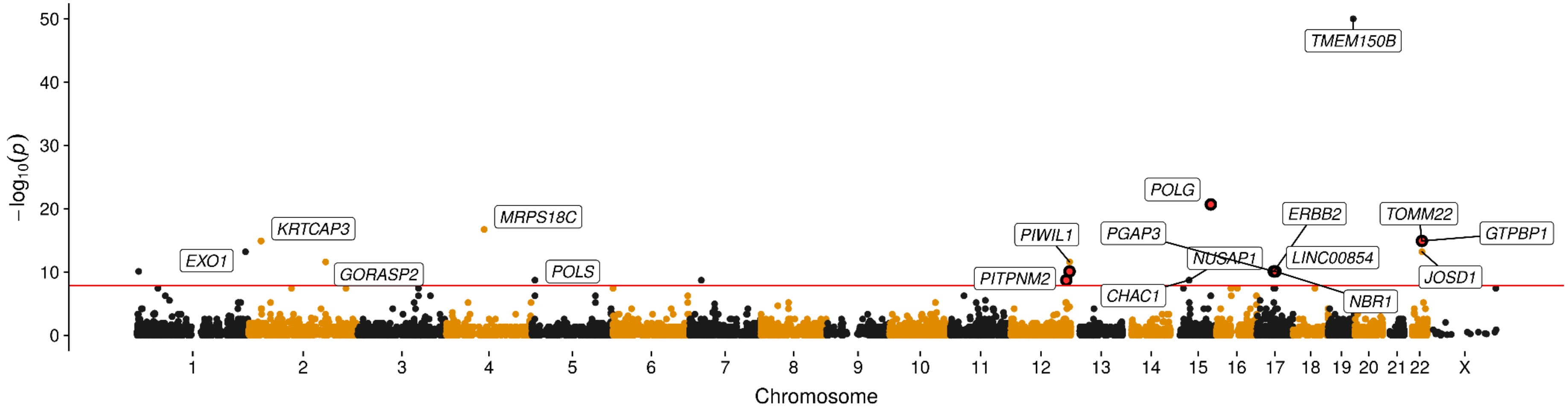

Anthropometric

Birth weight

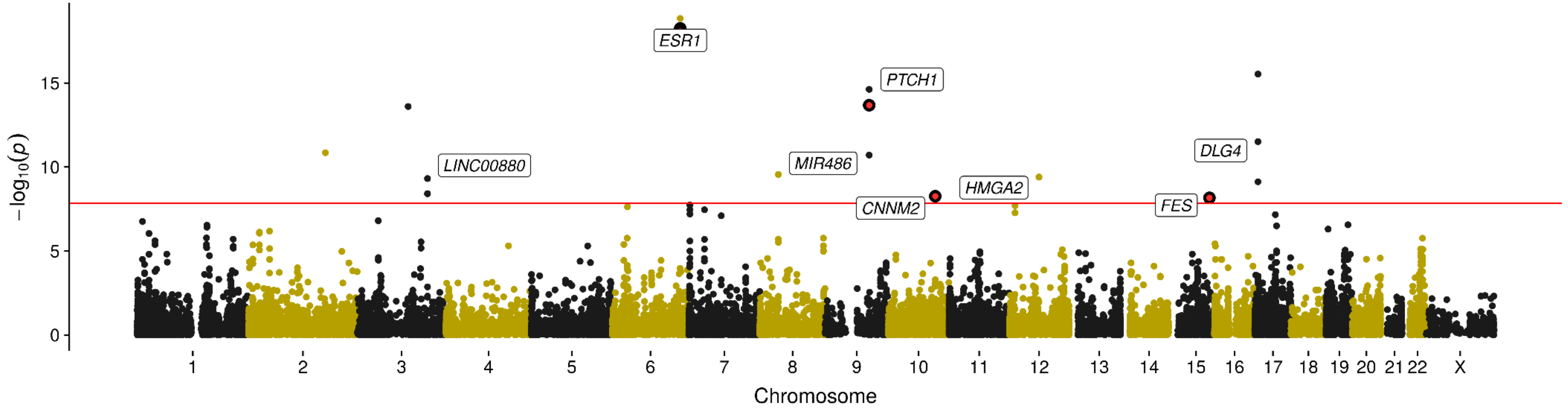

Body mass Index

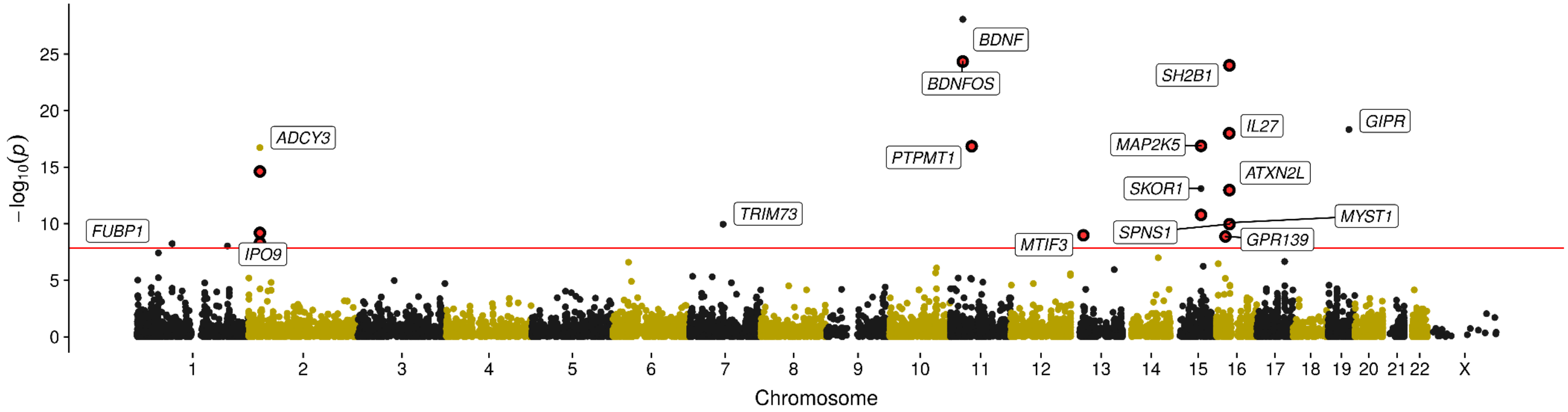

Childhood obesity

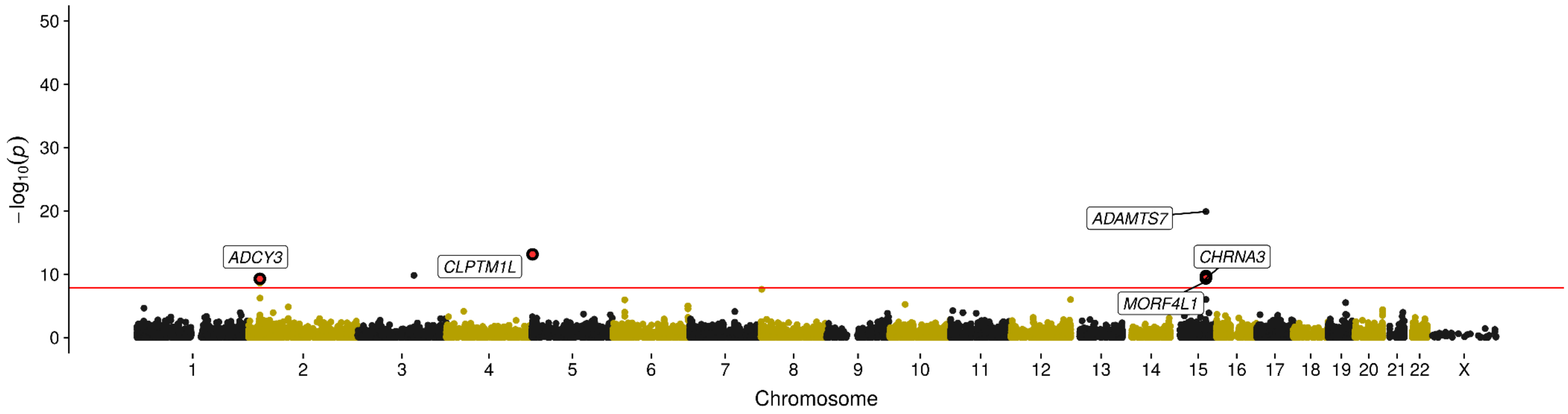

Extreme height

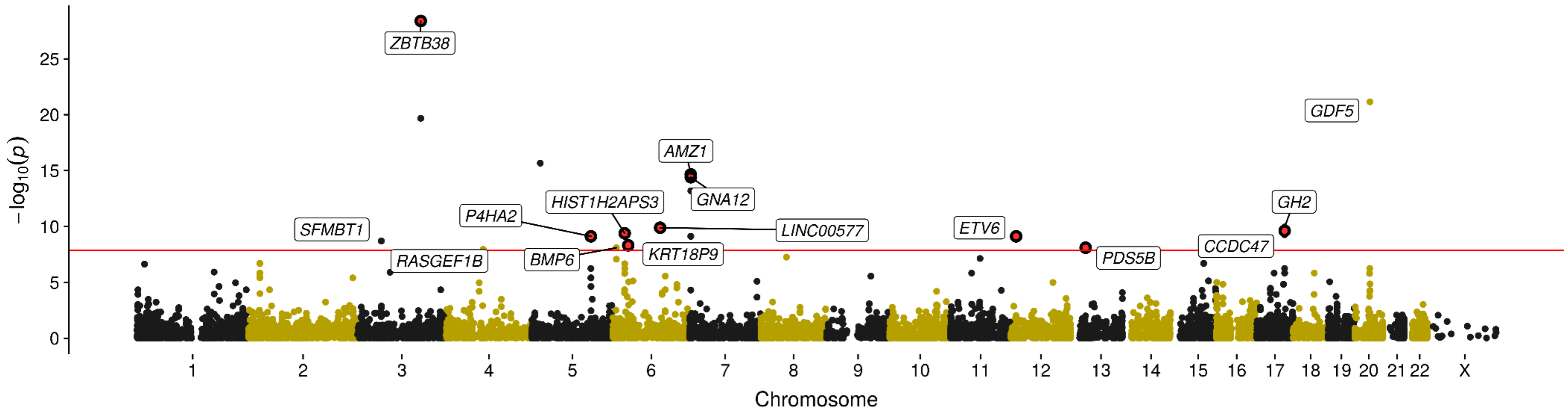

Height

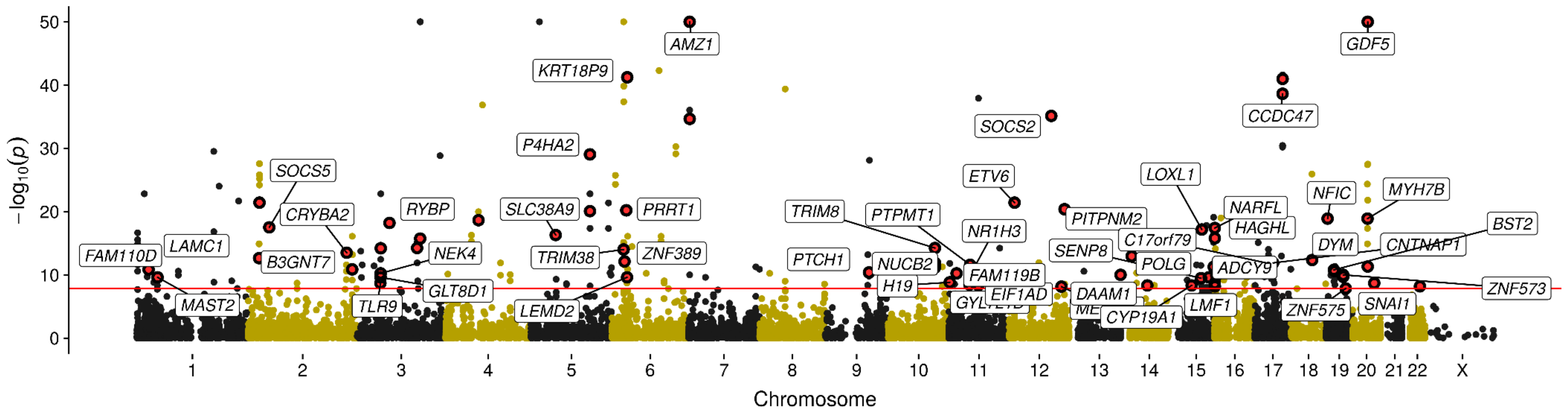

50
40
30
20
10
0
−log10(p)
AMZ1
GDF5
KRT18P9
CCDC47
SOCS2
P4HA2
SOCS5
ETV6
LOXL1
MYH7B
NFIC
CRYBA2
RYBP
SLC38A9
PRRT1
TRIM8
PTPMT1
NARFL
PITPNM2
BST2
HAGHL
NR1H3
C17orf79
DYM
CNTNAP1
LAMC1
FAM110D
NEK4
TRIM38
ZNF389
SENP8
B3GNT7
PTCH1
NUCB2
POLG
ADCY9
FAM119B
ZNF573
GLT8D1
H19
EIF1AD
DAAM1
MAST2
TLR9
LMF1
SNAI1
LEMD2
ZNF575
CYP19A1
1
2
3
4
5
6
7
8
9
10
11
12
13
14
15
16
17
18
19
20
21
22
X
Chromosome

Hip circumference

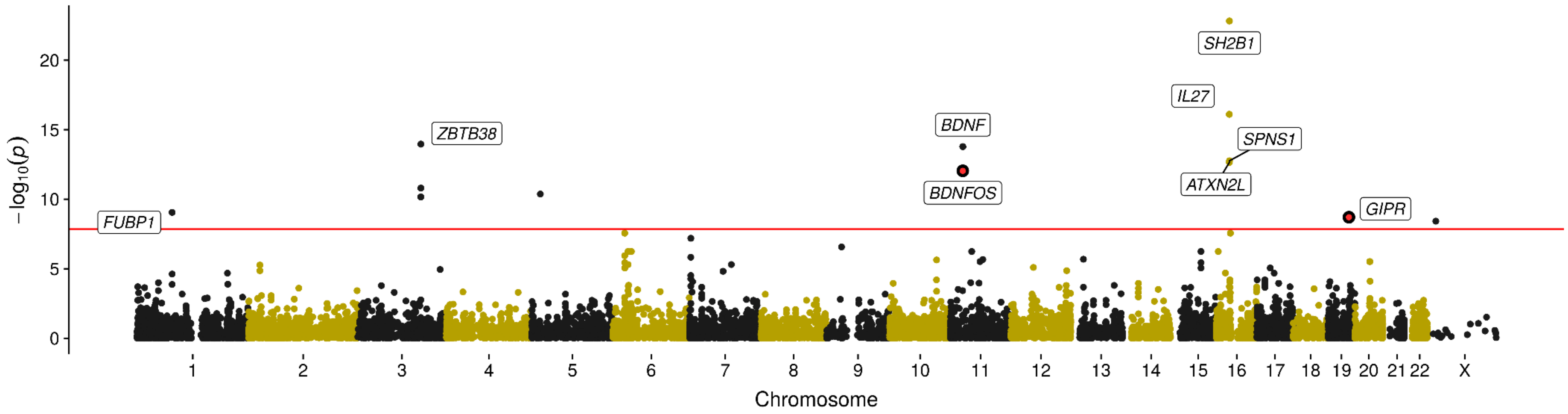

Obesity

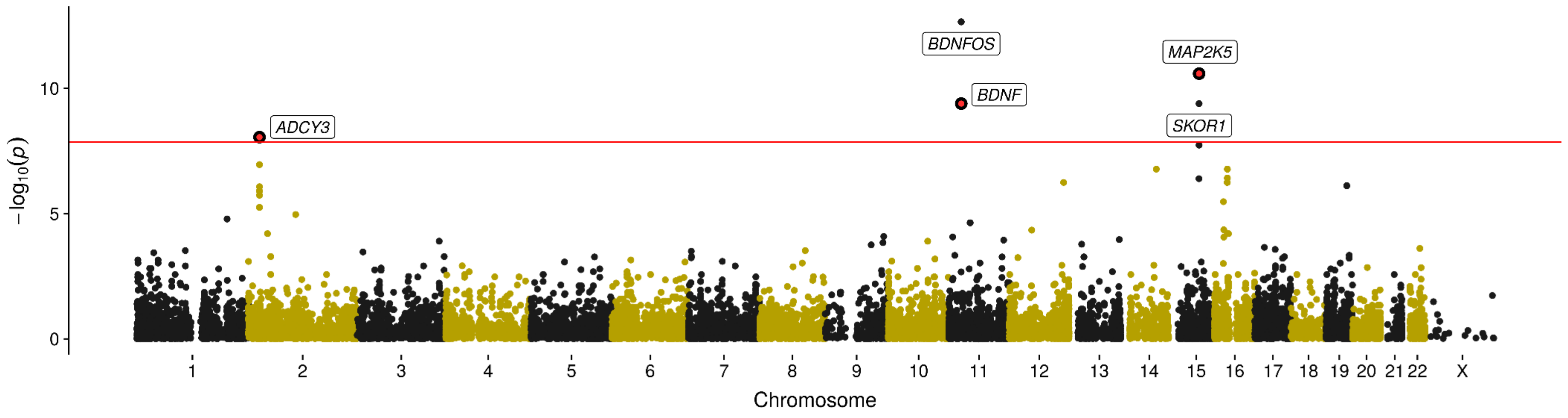
BDNFOS
MAP2K5
BDNF
ADCY3
SKOR1
10
5
0
−log10(p)
1
2
3
4
5
6
7
8
9
10
11
12
13
14
15
16
17
18
19
20
21
22
X
Chromosome

Overweight

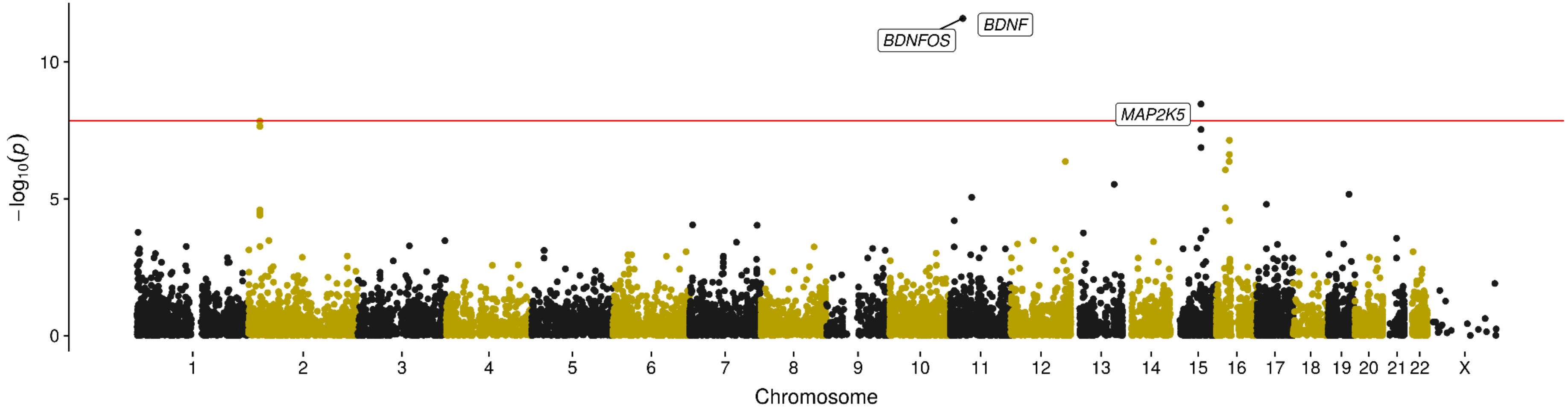

BDNFOS
BDNF
MAP2K5
10
5
0
−log10(p)
1
2
3
4
5
6
7
8
9
10
11
12
13
14
15
16
17
18
19
20
21
22
X
Chromosome

Sitting height ratio

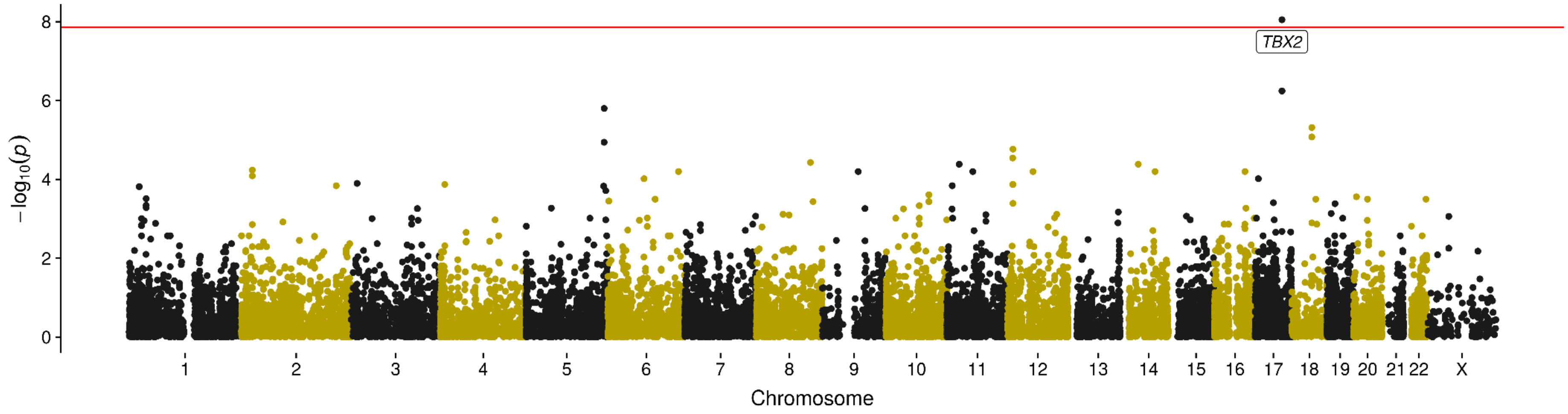

Waist-to-hip ratio

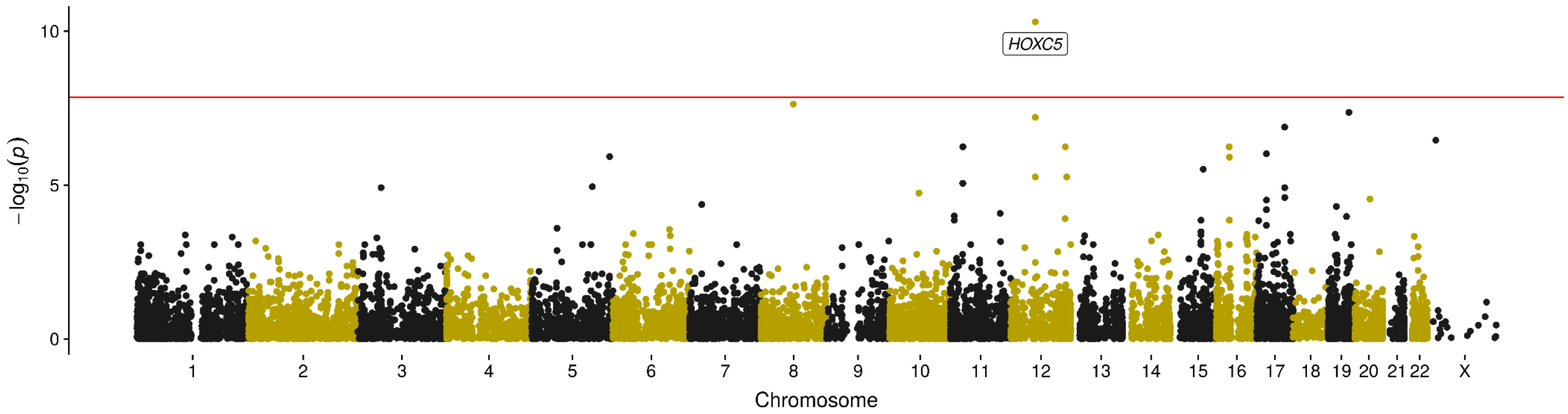

Waist circumference

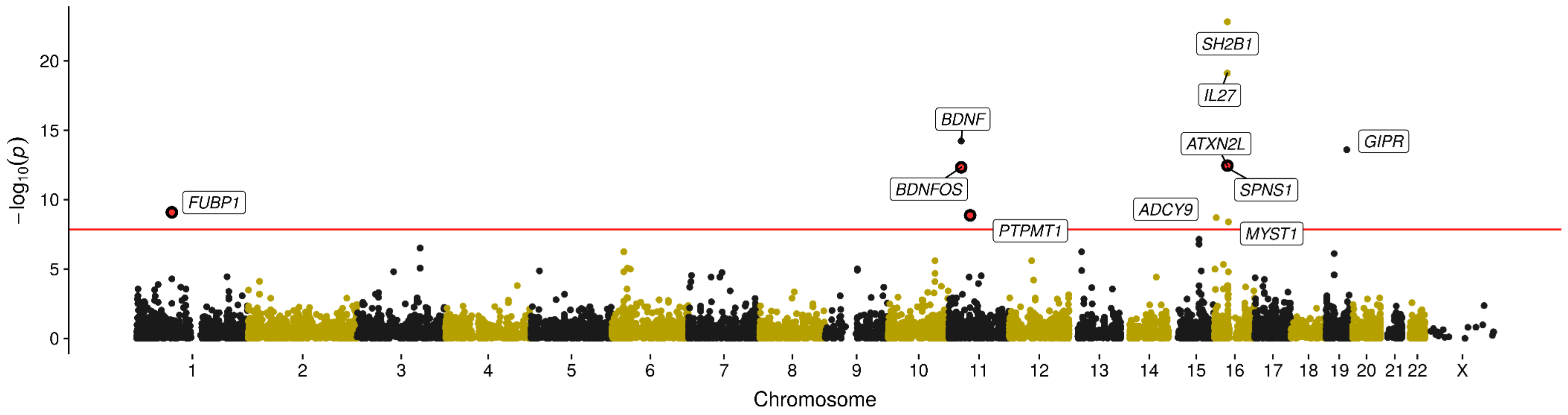

Weight

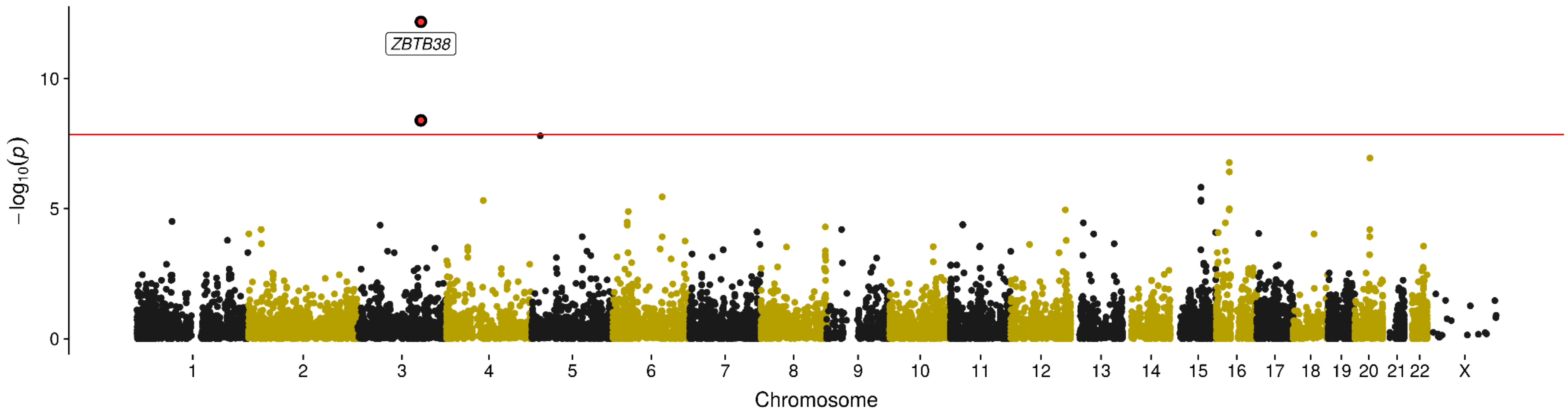

ZBTB38
10
5
0
−log10(p)
1
2
3
4
5
6
7
8
9
10
11
12
13
14
15
16
17
18
19
20
21
22
X
Chromosome

Autoimmune / inflammatory

Asthma

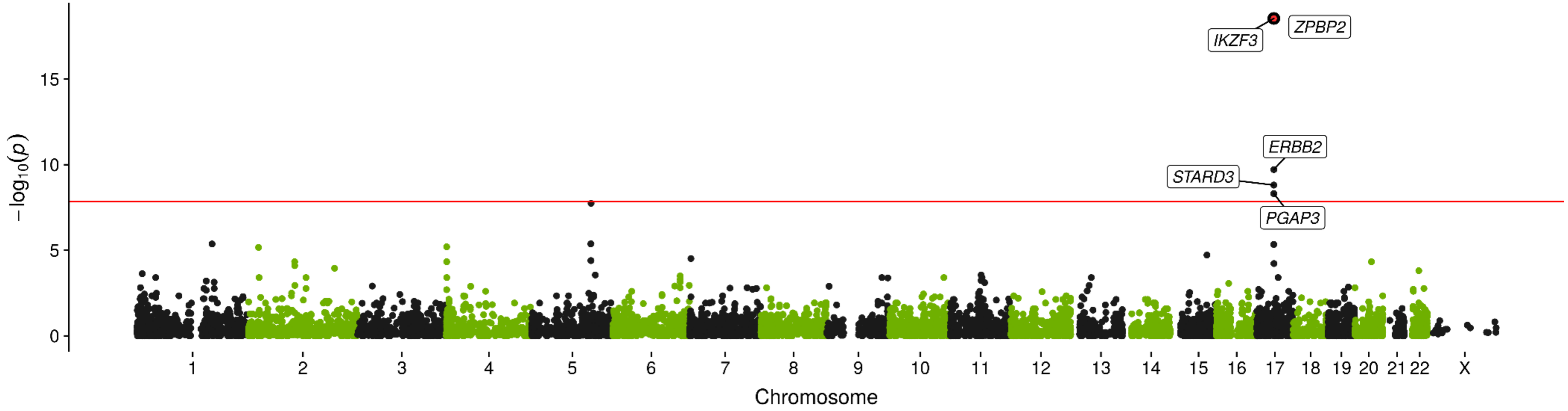

Celiac disease

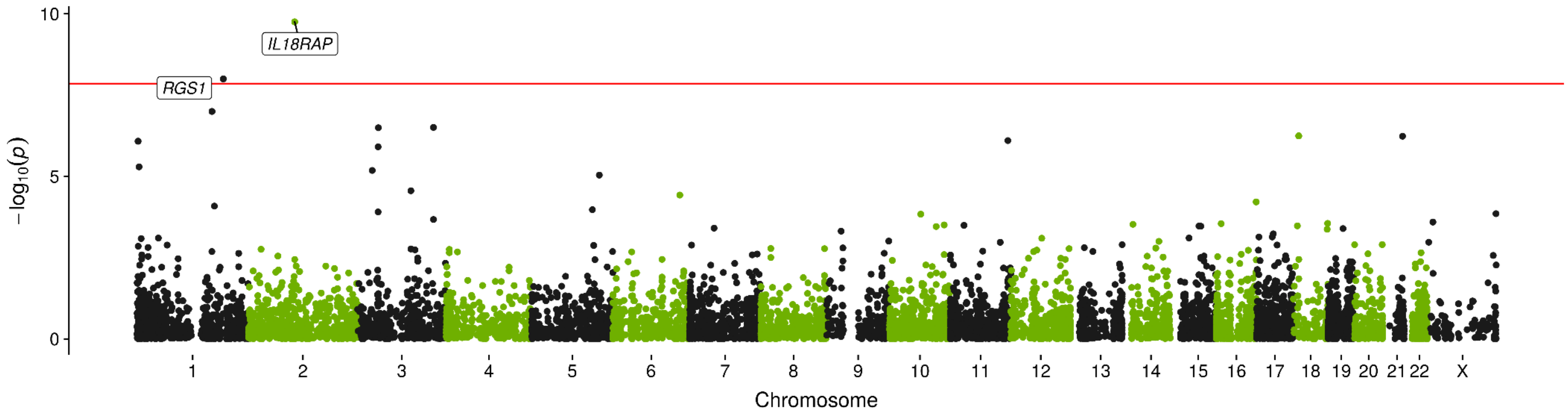

Crohn's disease

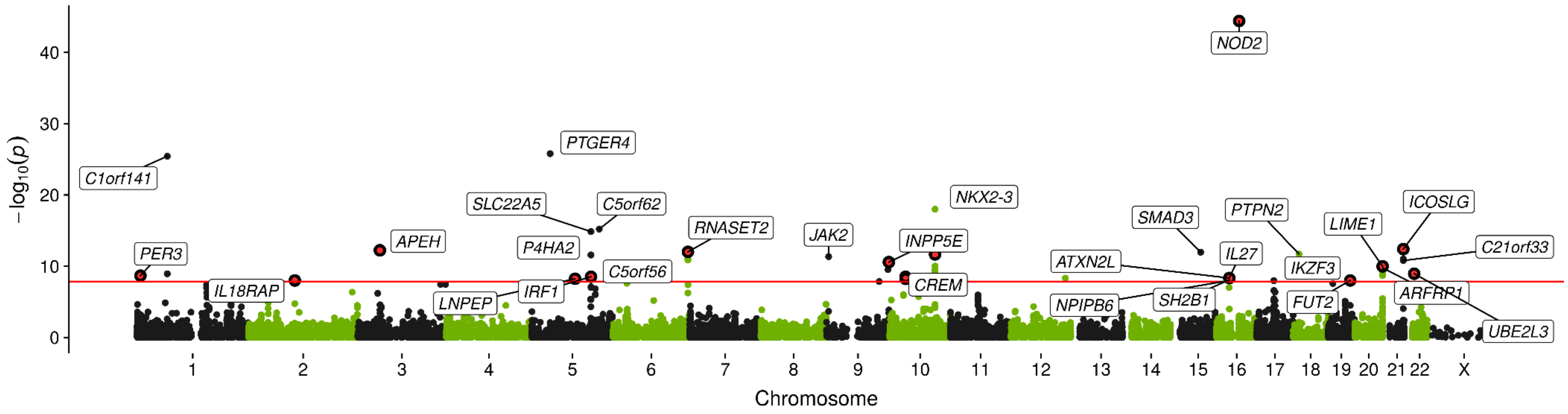

Eczema

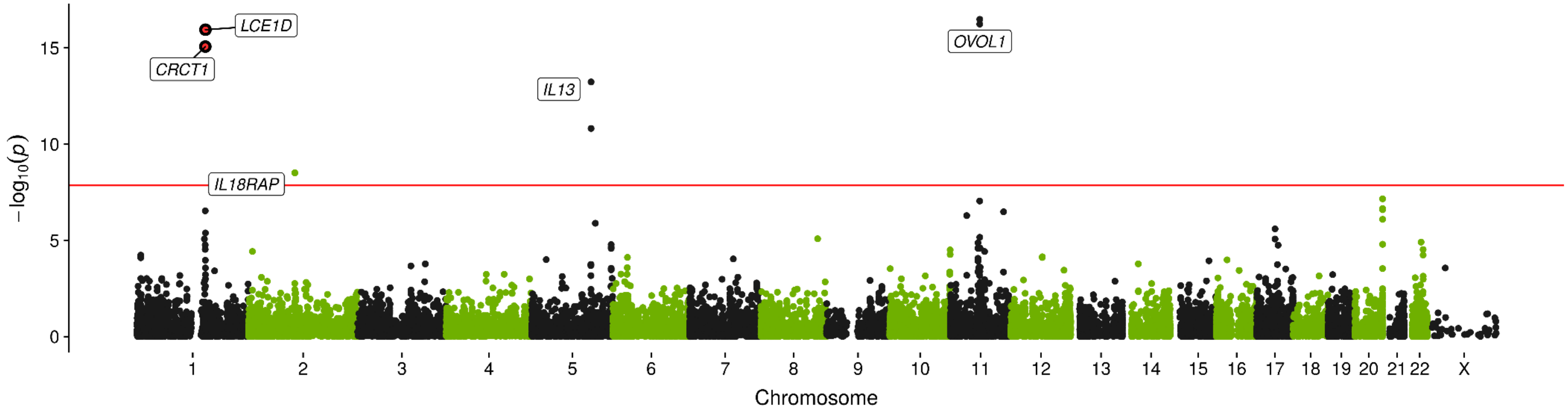

LCE1D
CRCT1
IL13
OVOL1
IL18RAP
−log10(p)
15
10
5
0
1
2
3
4
5
6
7
8
9
10
11
12
13
14
15
16
17
18
19
20
21
22
X
Chromosome

Inflammatory bowel disease

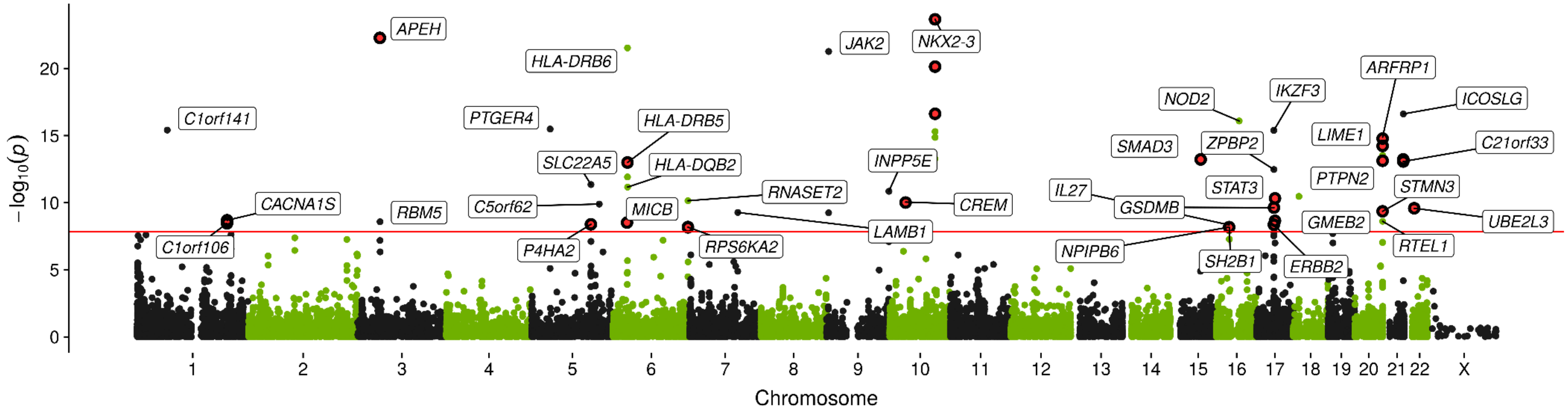

Multiple sclerosis

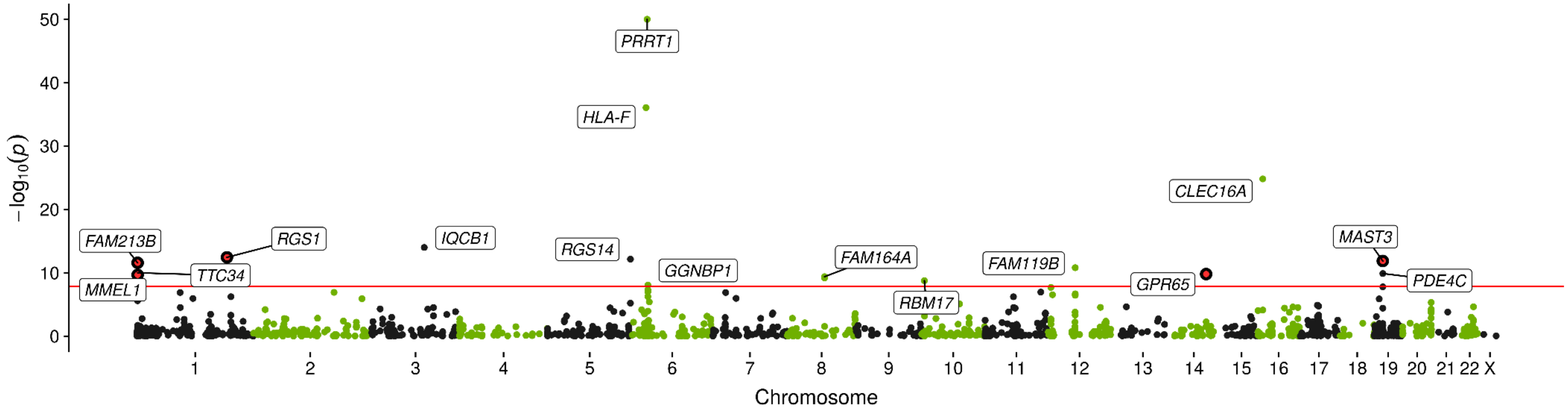

Rheumatoid arthritis

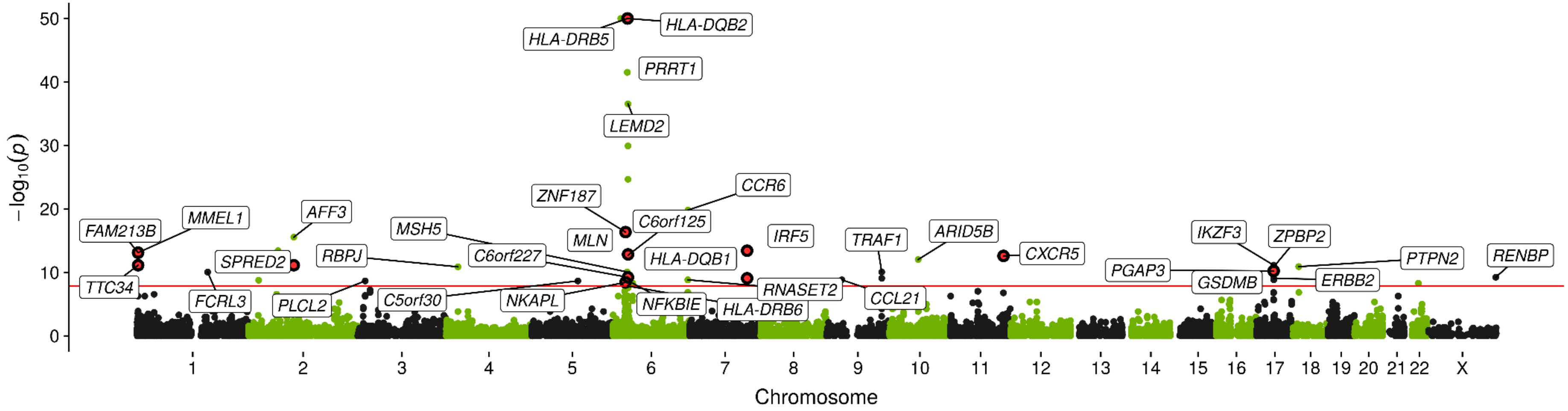

Ulcerative colitis

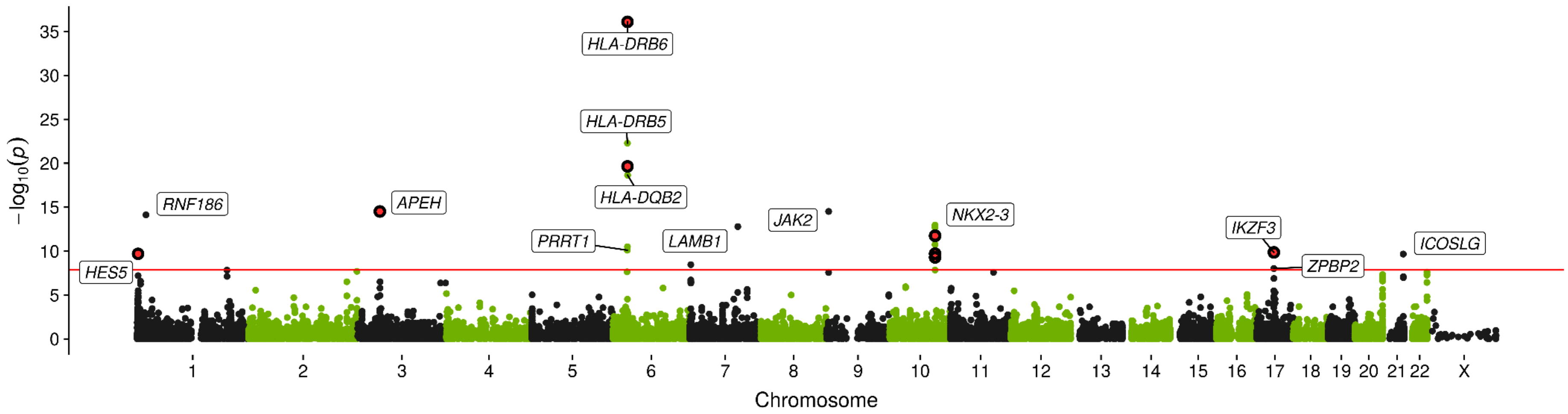

Education

Years of Schooling

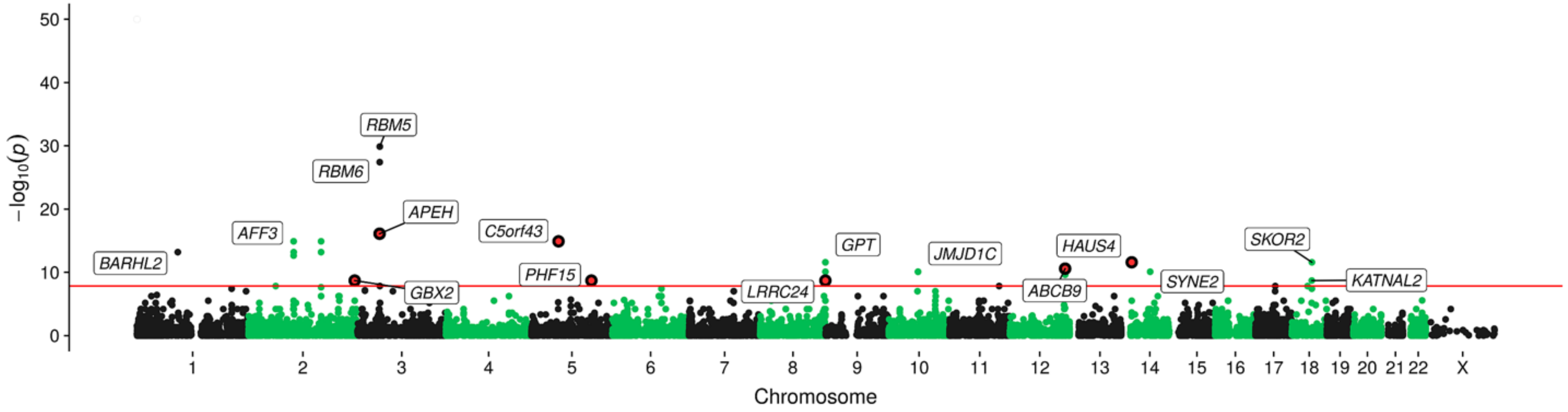

Cardiovascular

Adiponectin

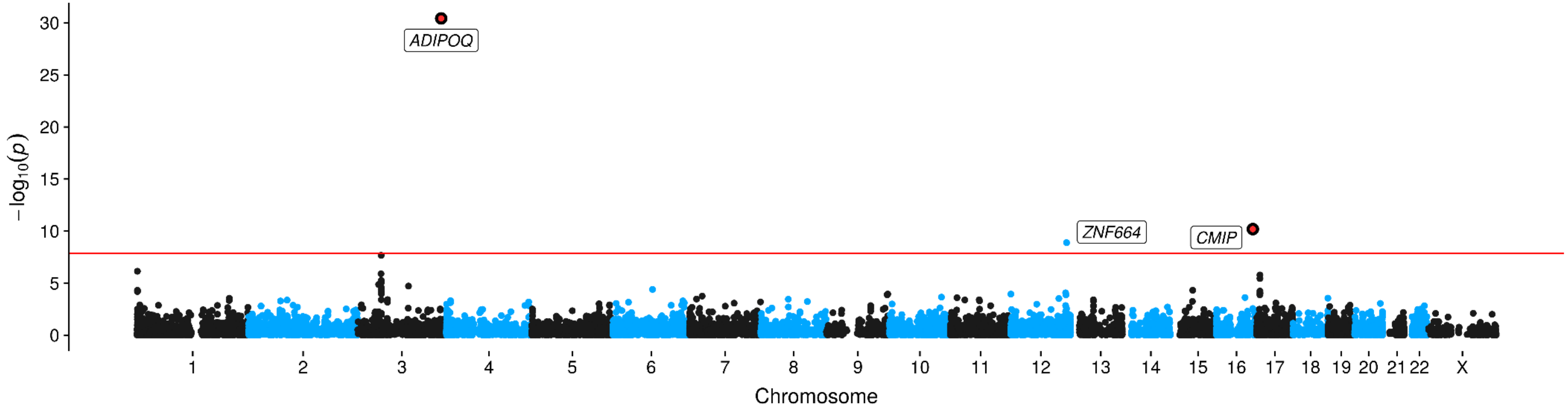

Coronary heart disease

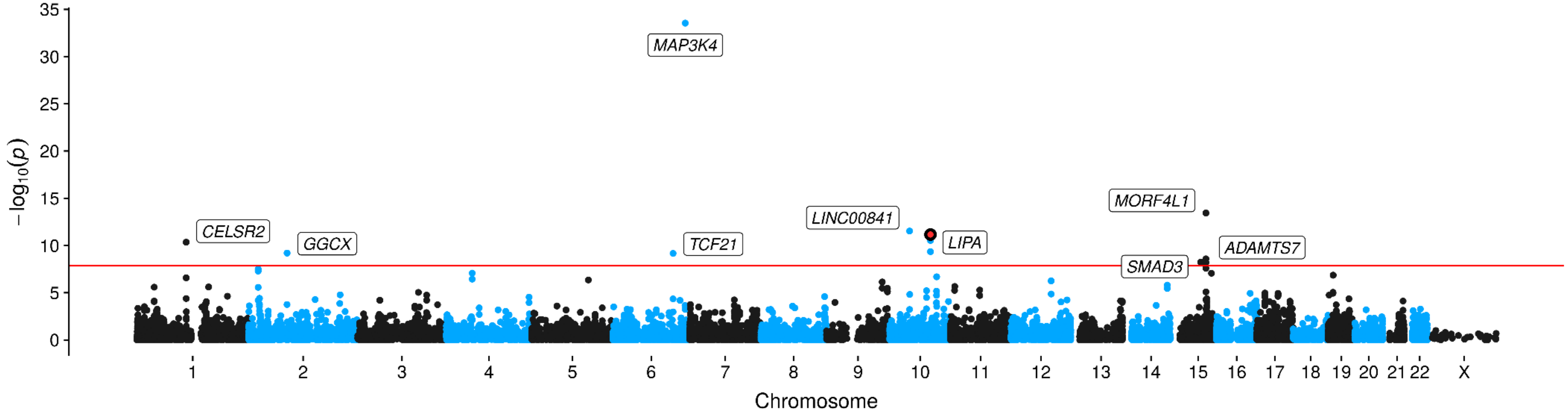

HDL Cholesterol

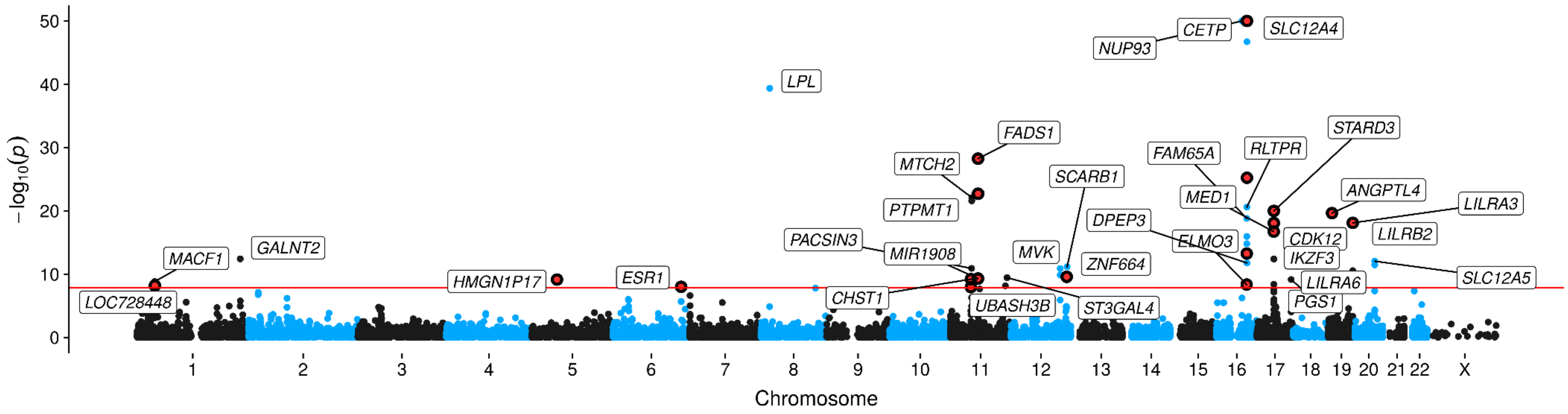

Heart rate

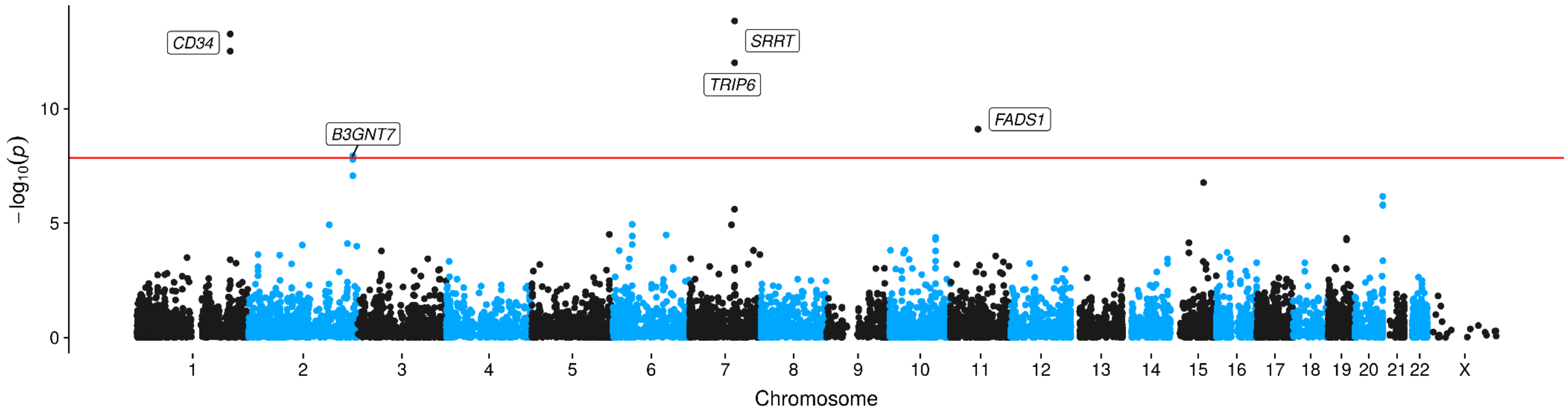
CD34
SRRT
TRIP6
FADS1
B3GNT7
−log10(p)
10
5
0
1
2
3
4
5
6
7
8
9
10
11
12
13
14
15
16
17
18
19
20
21
22
X
Chromosome

LDL Cholesterol

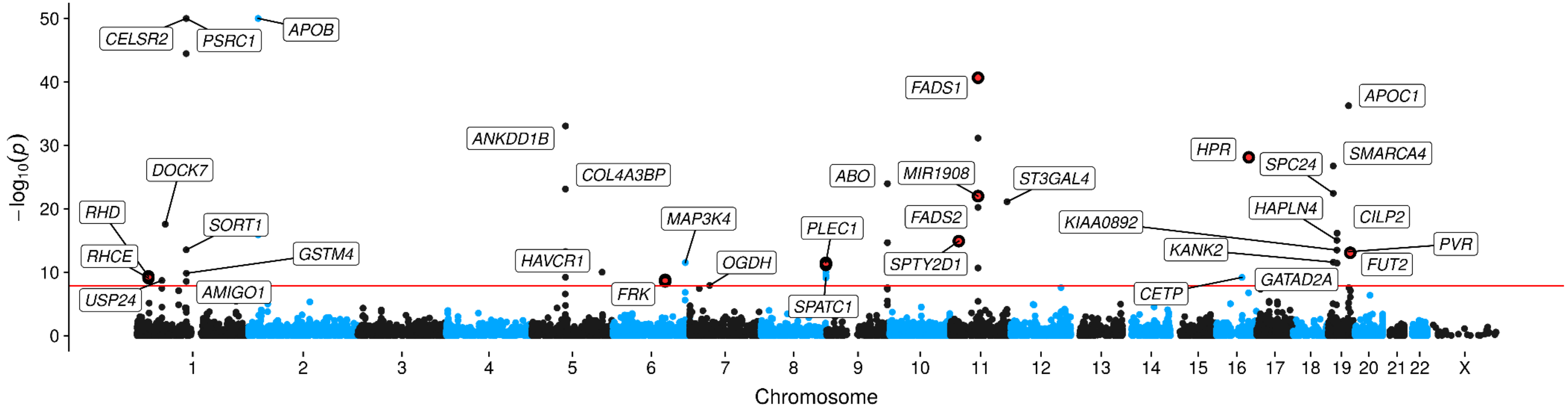

Myocardial Infarction

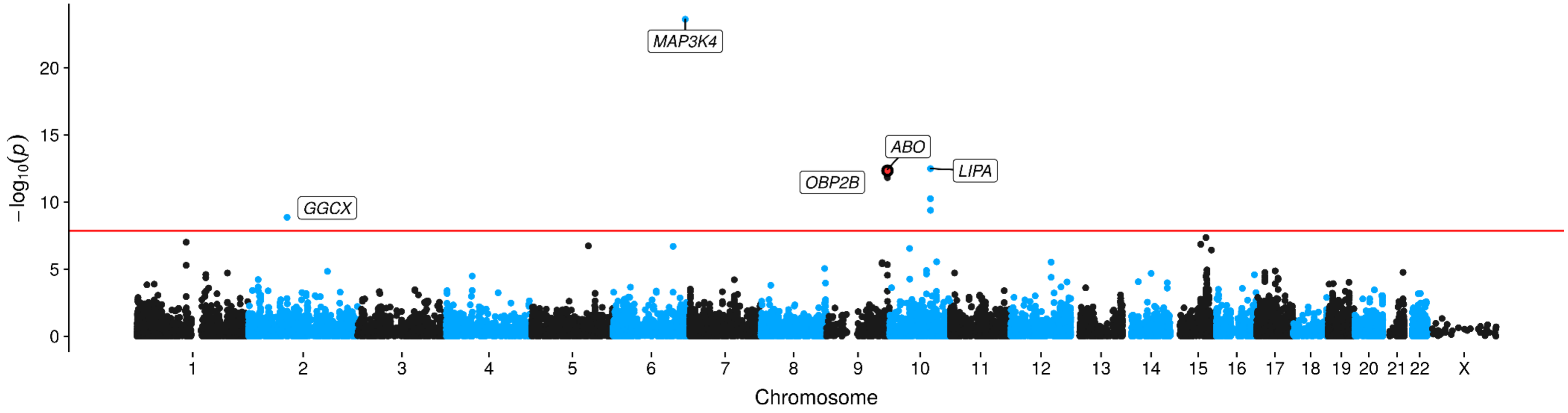

Total Cholesterol

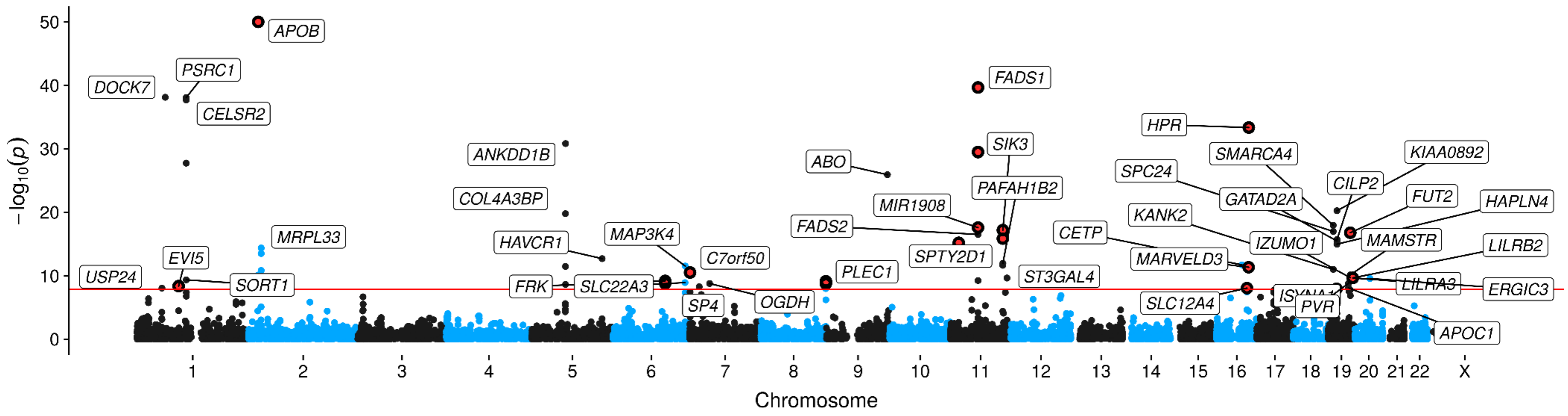

Triglycerides

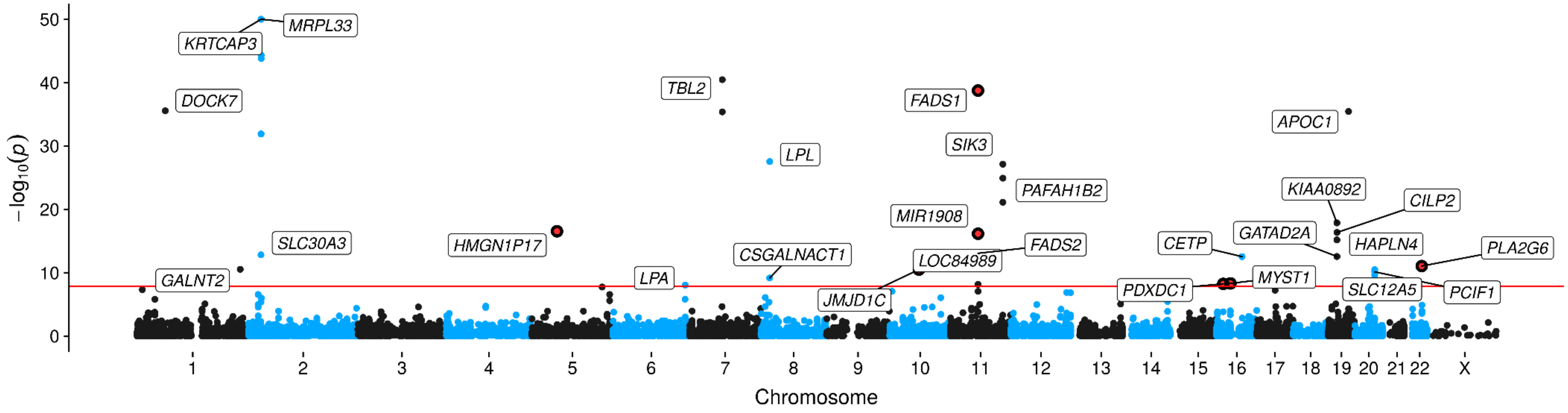

Glycemic

Fasting proinsulin

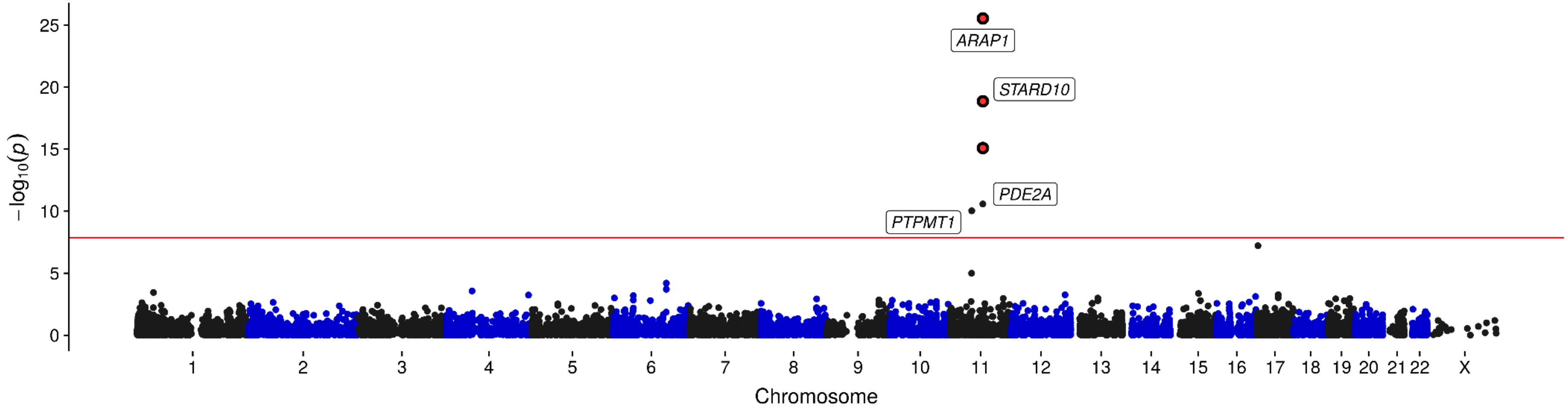

HOMA-B

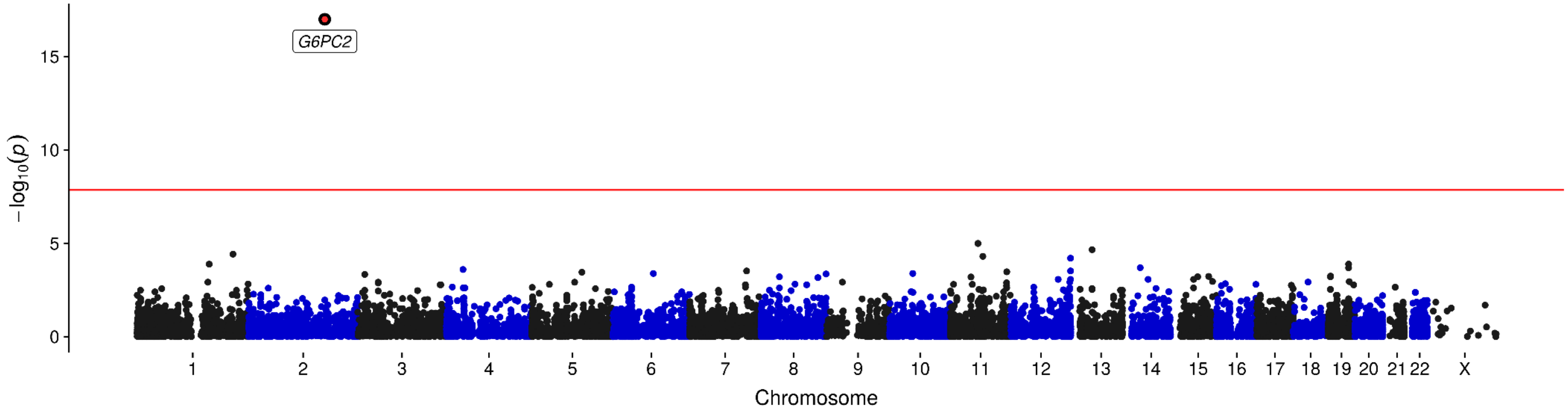

G6PC2
15
10
5
0
$-\log_{10}(p)$
1 2 3 4 5 6 7 8 9 10 11 12 13 14 15 16 17 18 19 20 21 22 X
Chromosome

HbA1C

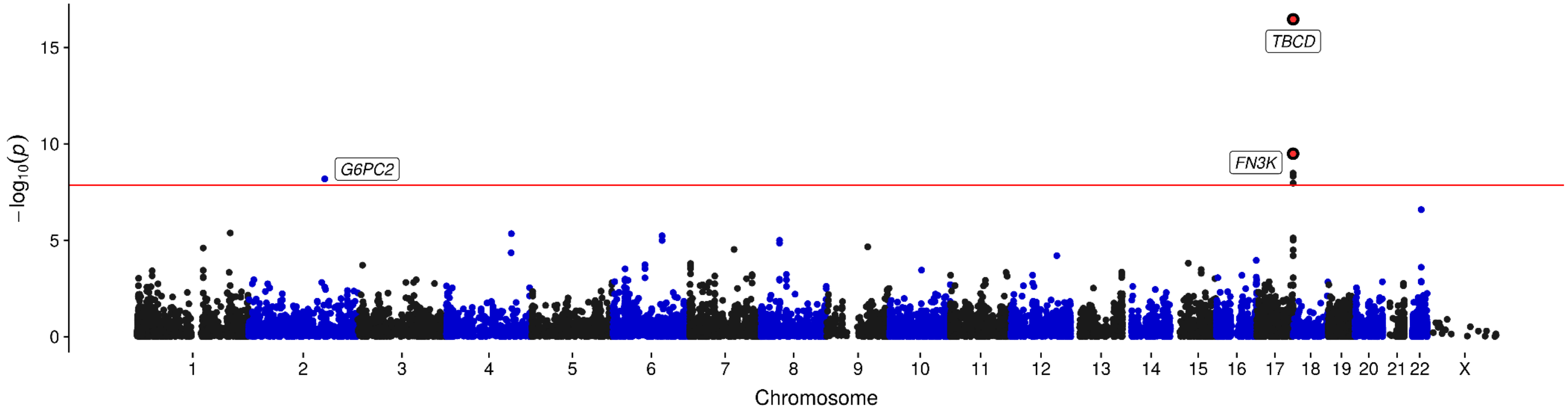

15
10
5
0
−log10(p)
TBCD
FN3K
G6PC2
1
2
3
4
5
6
7
8
9
10
11
12
13
14
15
16
17
18
19
20
21
22
X
Chromosome

Type 2 diabetes

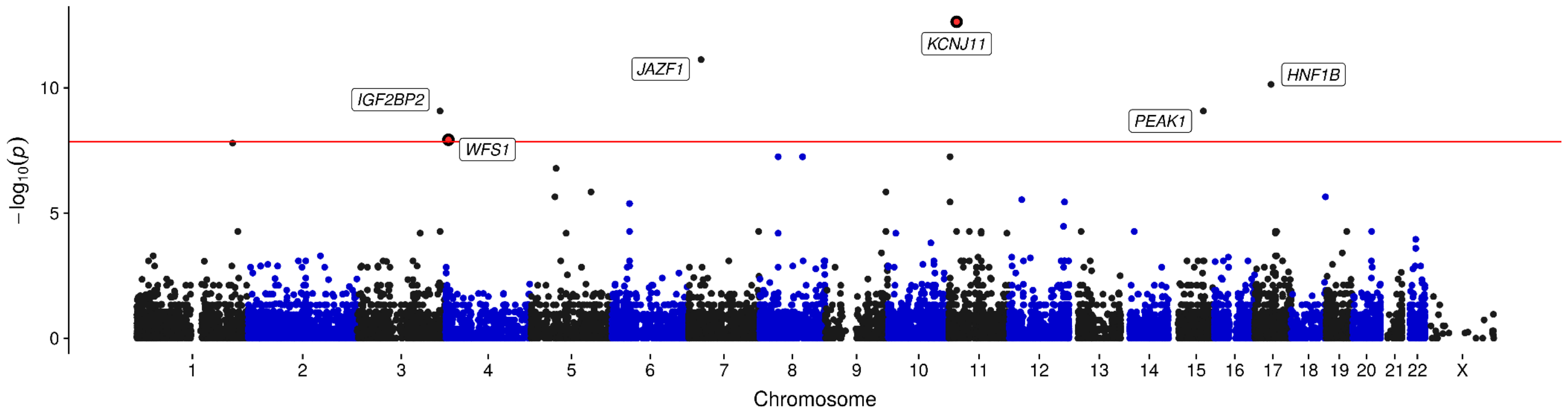

Haemotological

Haemoglobin concentration

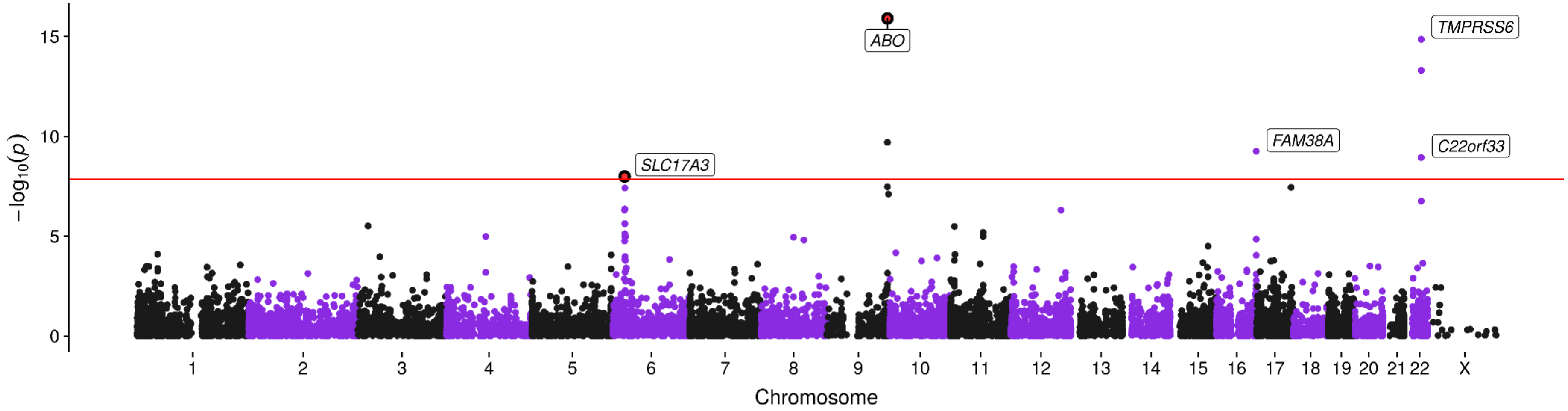

Mean cell haemoglobin

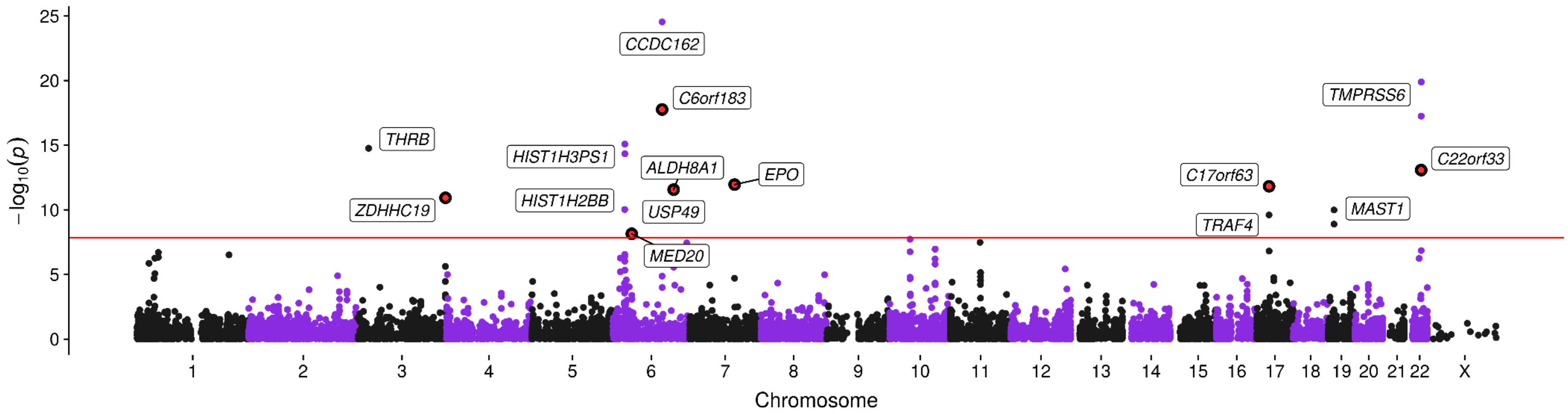

Mean cell volume

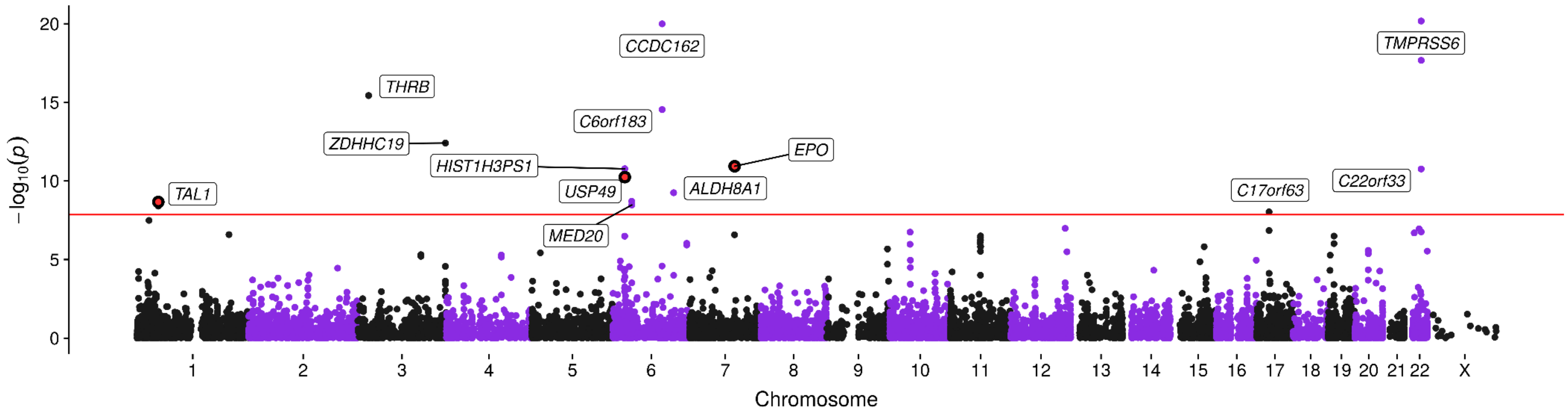

Mean platelet volume

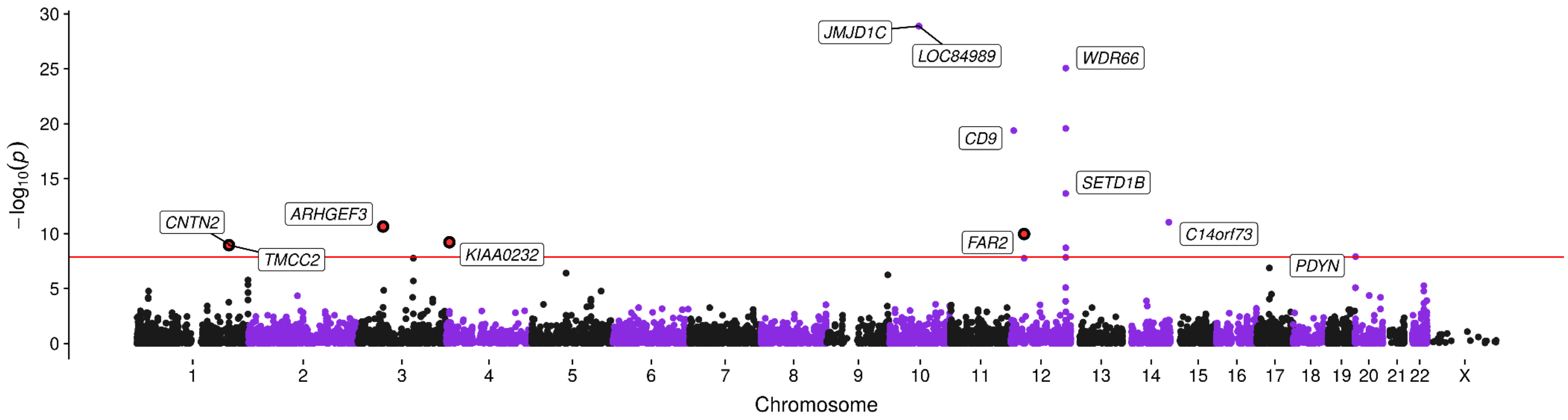

Packed cell volume

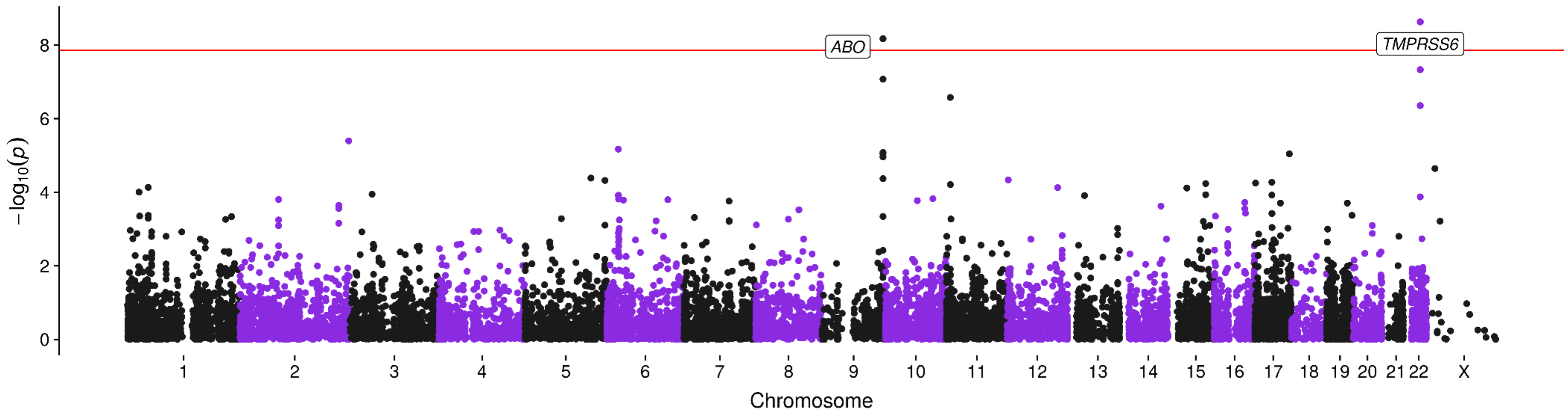

Platelet count

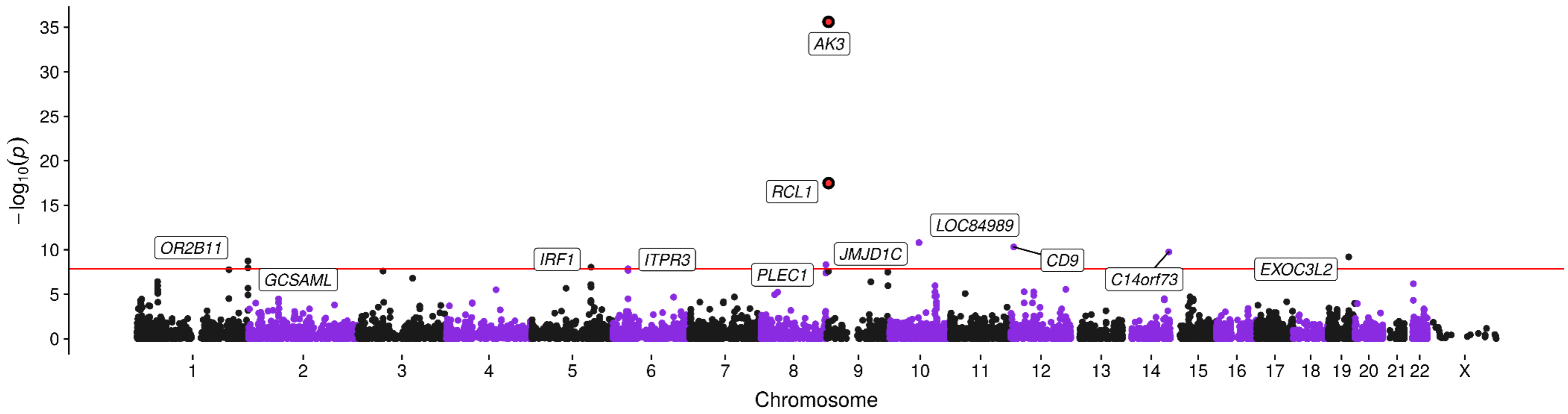

Red blood cell count

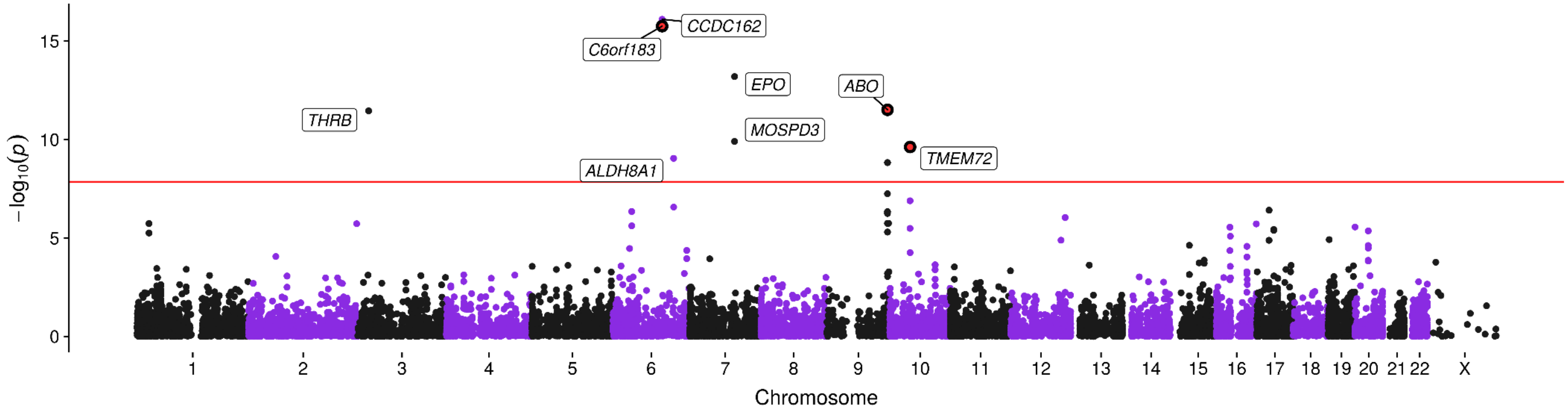

Kidney

Chronic kidney disease

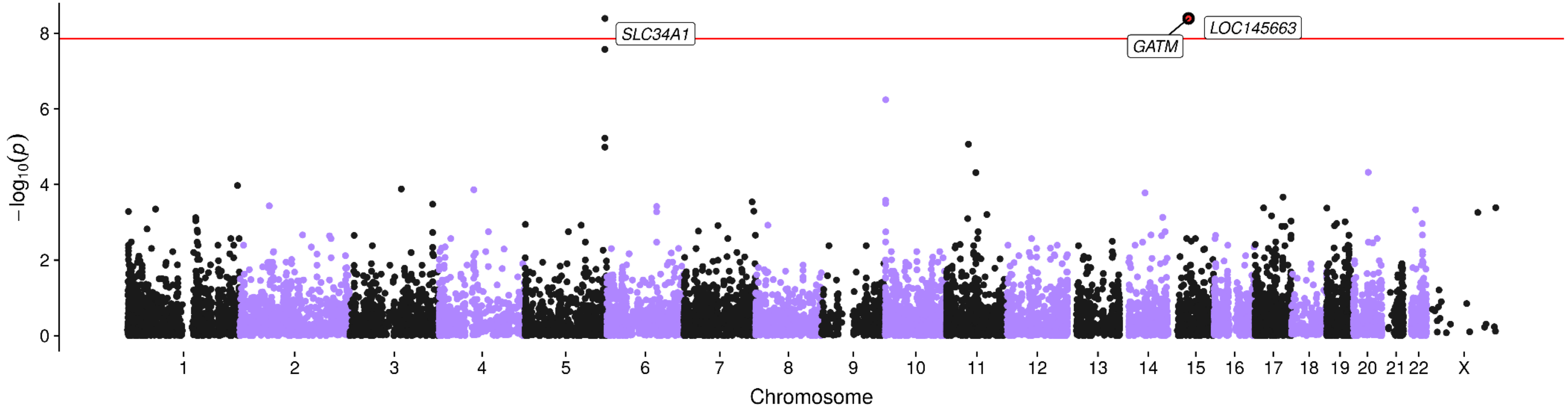

Microalbuminuria

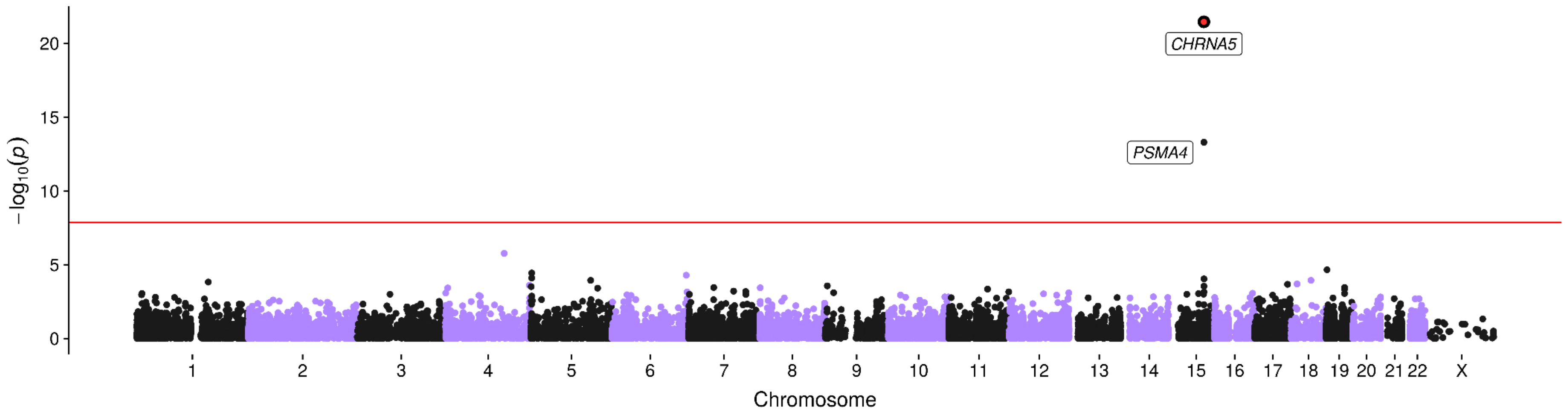

Serum creatinine

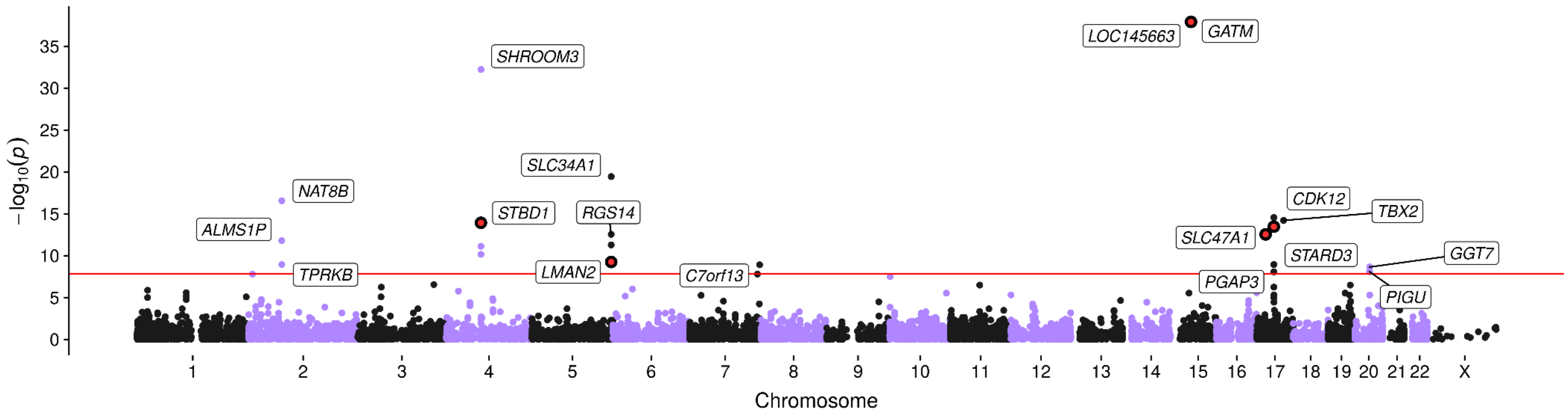

Serum cystatin C

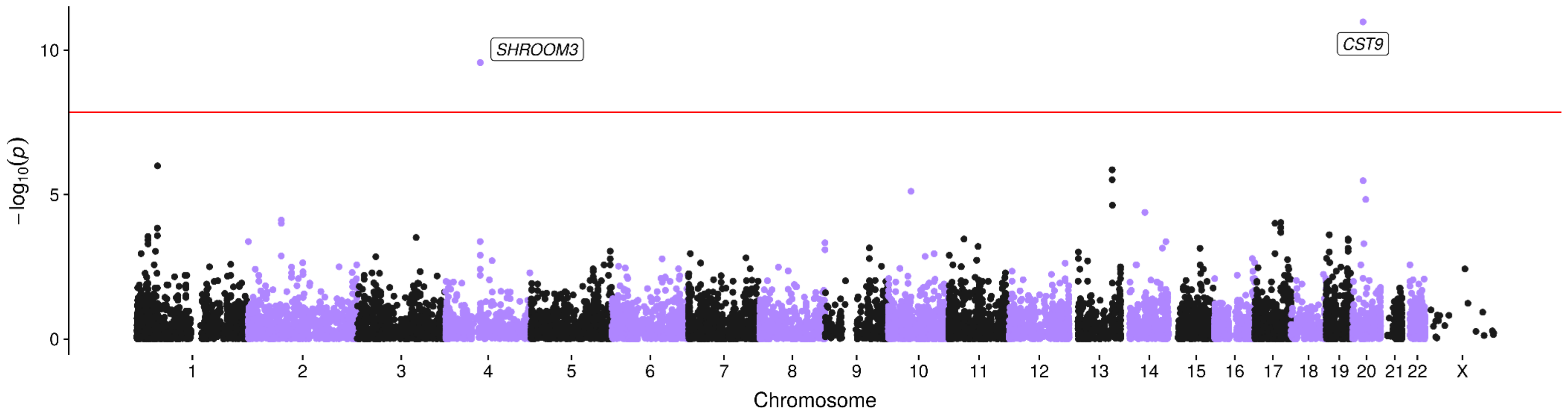

Psychiatric / neurological

Alzheimer's disease

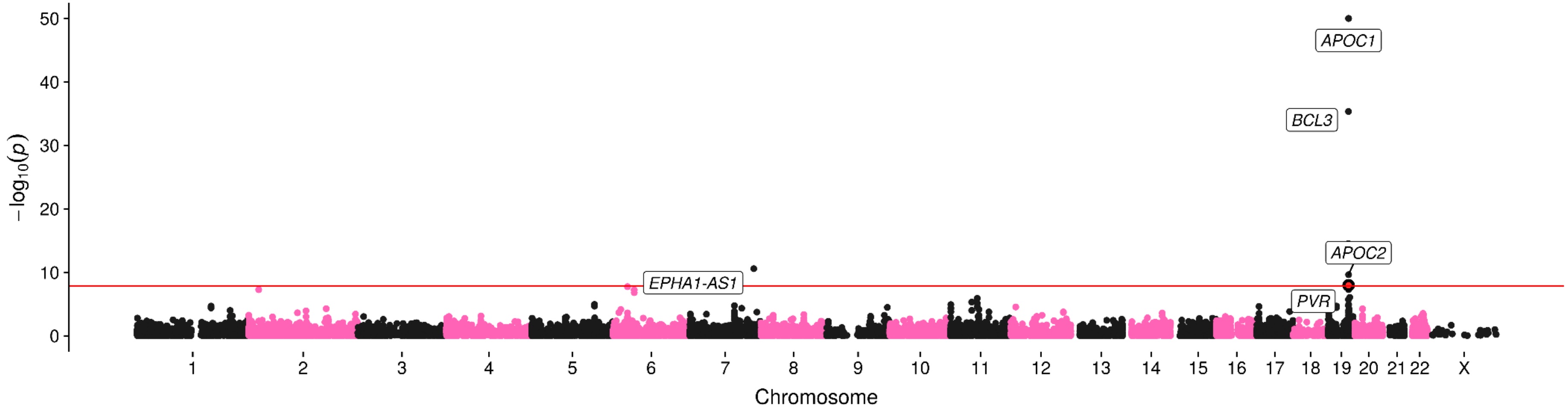

Anorexia nervosa

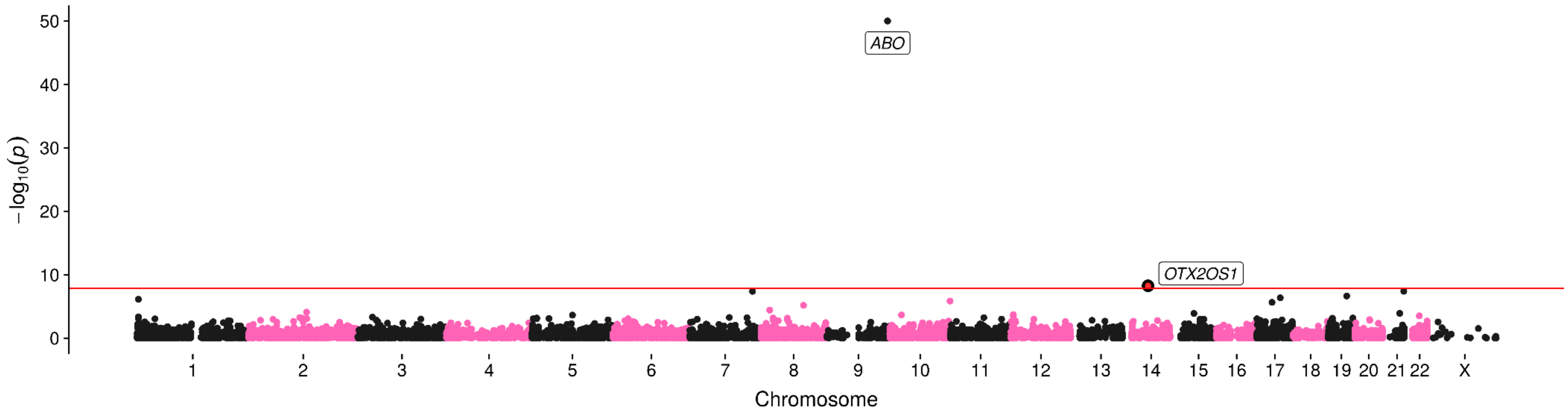

PGC cross-disorder traits

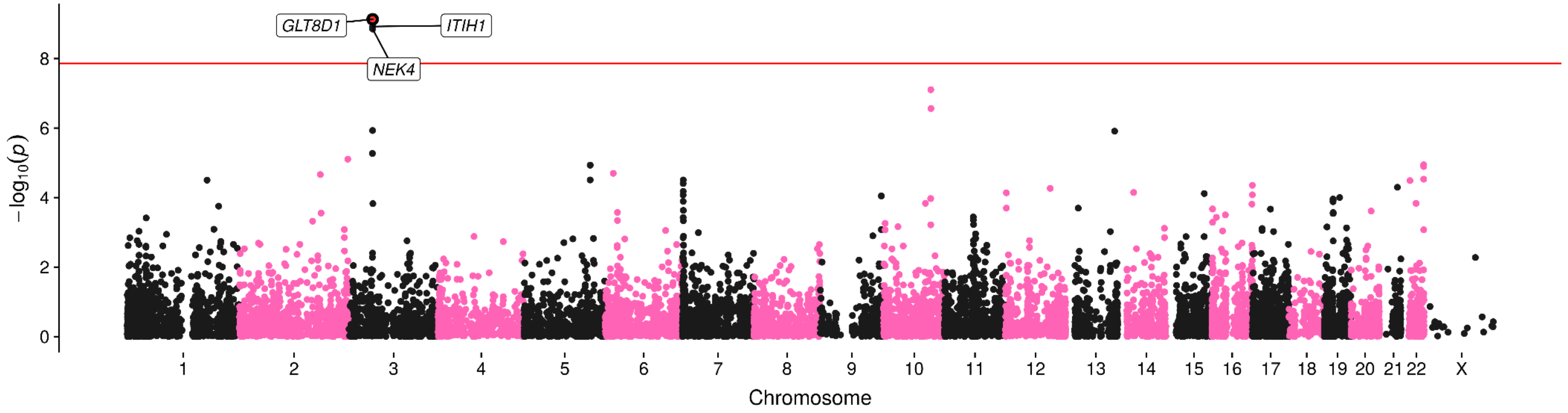

Putamen volume

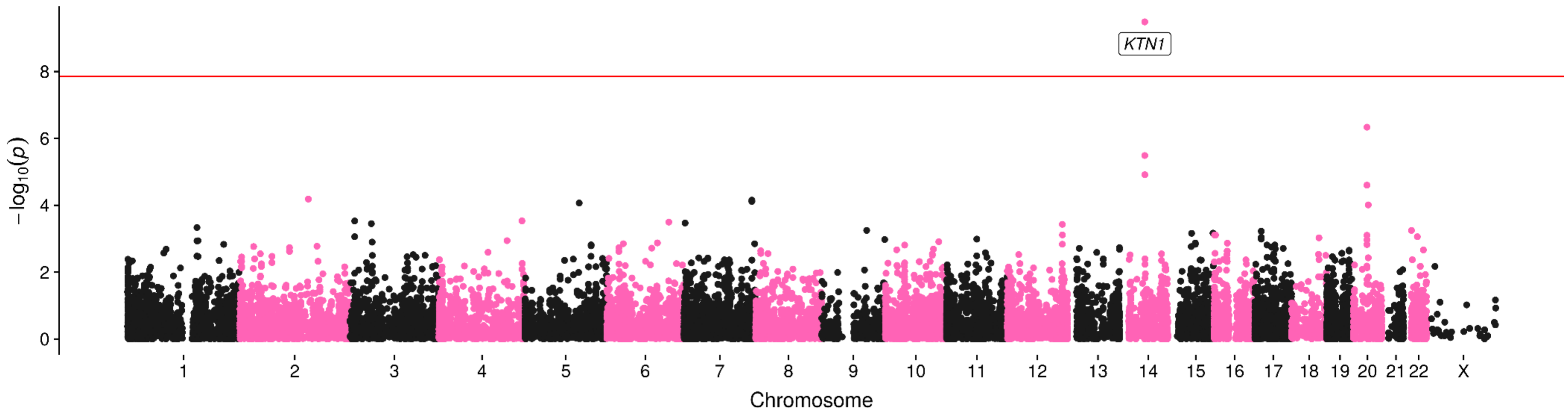

Neuroticism

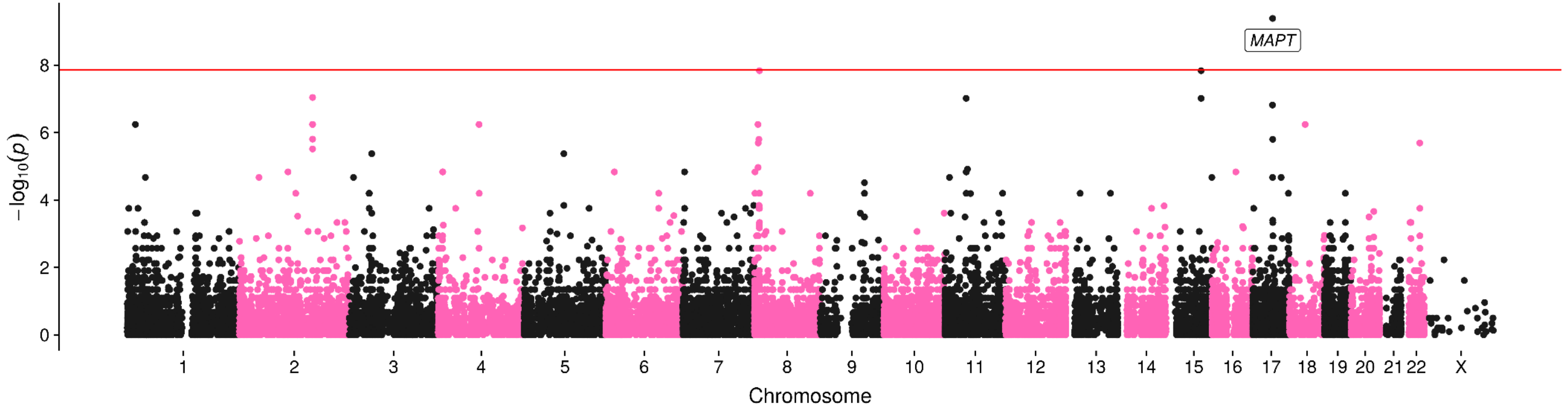

Schizophrenia

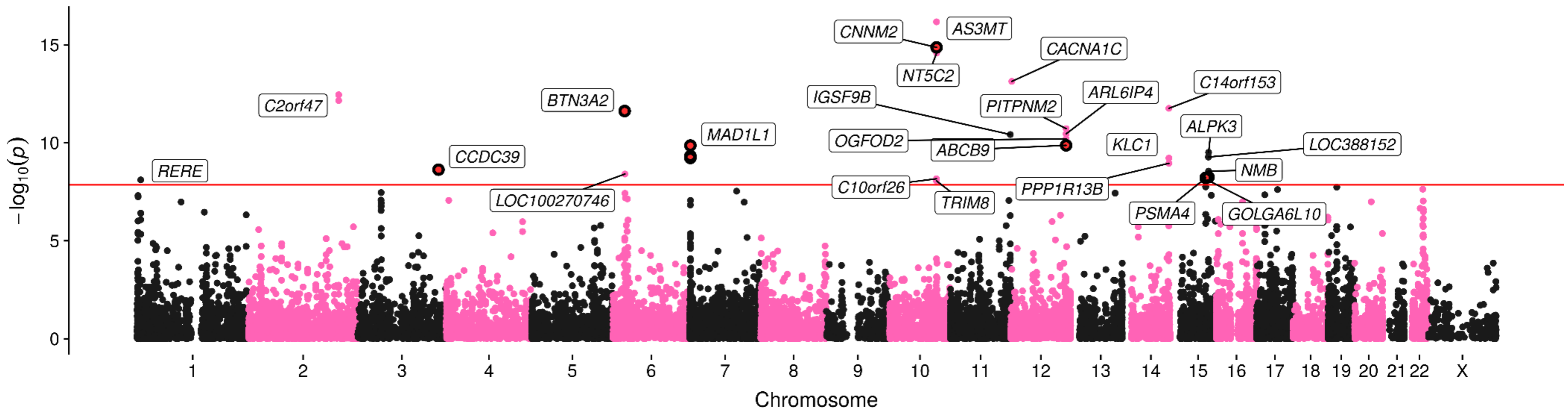

Other

Cigarettes smoked per day

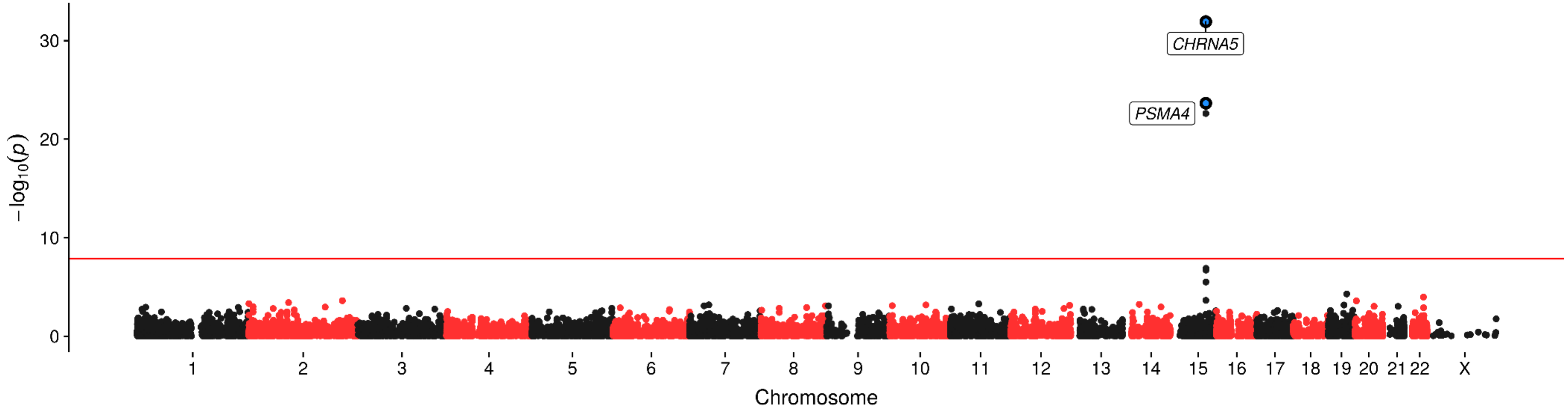

Copper

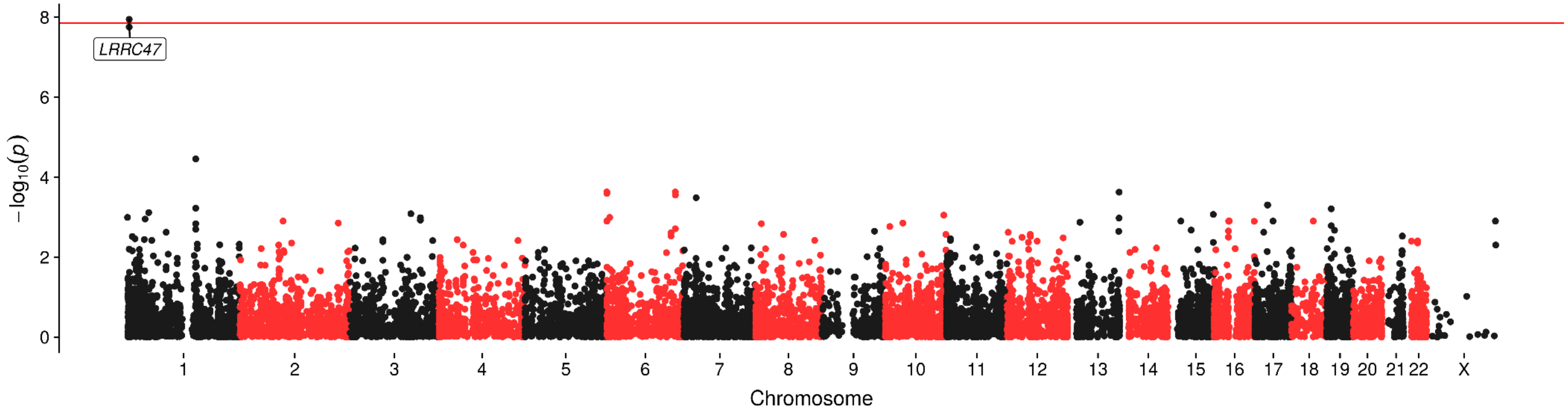

LRRC47
−log10(p)
8
6
4
2
0
1
2
3
4
5
6
7
8
9
10
11
12
13
14
15
16
17
18
19
20
21
22
X
Chromosome

Iron

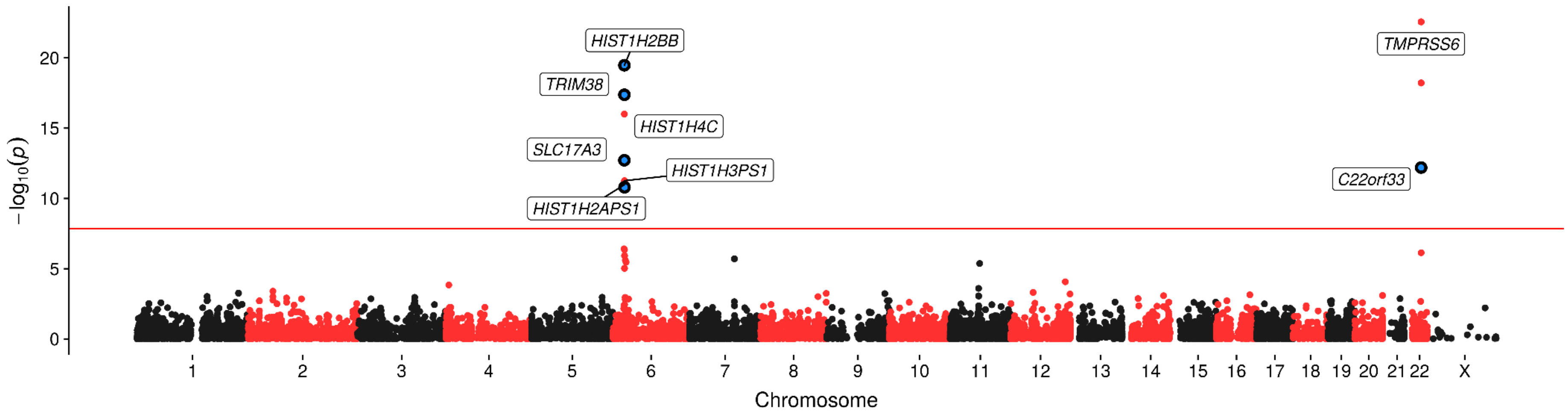

HIST1H2BB
TRIM38
HIST1H4C
SLC17A3
HIST1H3PS1
HIST1H2APS1
TMPRSS6
C22orf33
20
15
10
5
0
$-\log_{10}(p)$
1
2
3
4
5
6
7
8
9
10
11
12
13
14
15
16
17
18
19
20
21
22
X
Chromosome

Lung adenocarcinoma

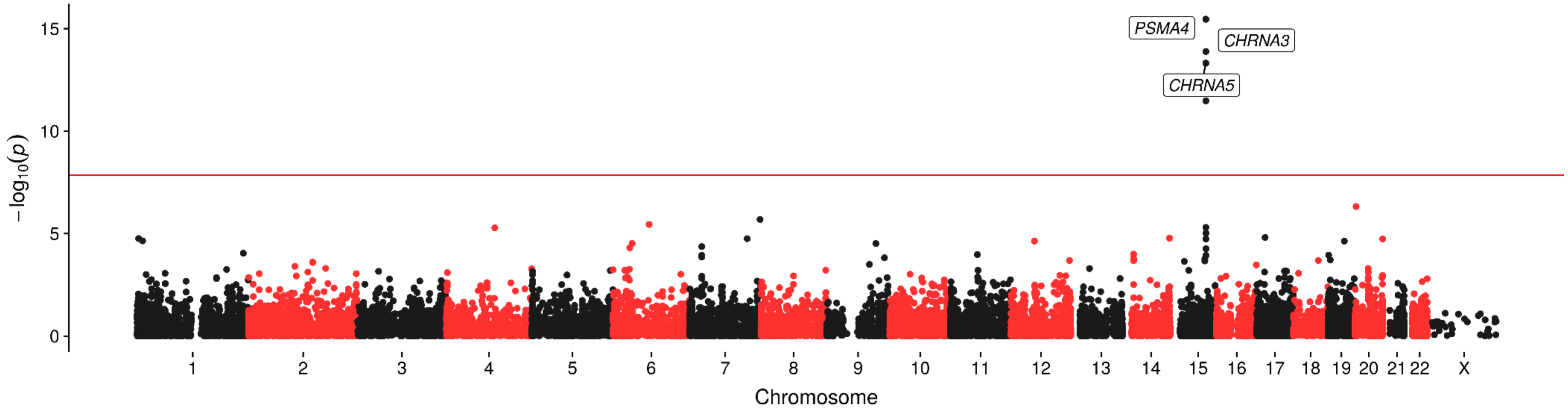

Lung cancer

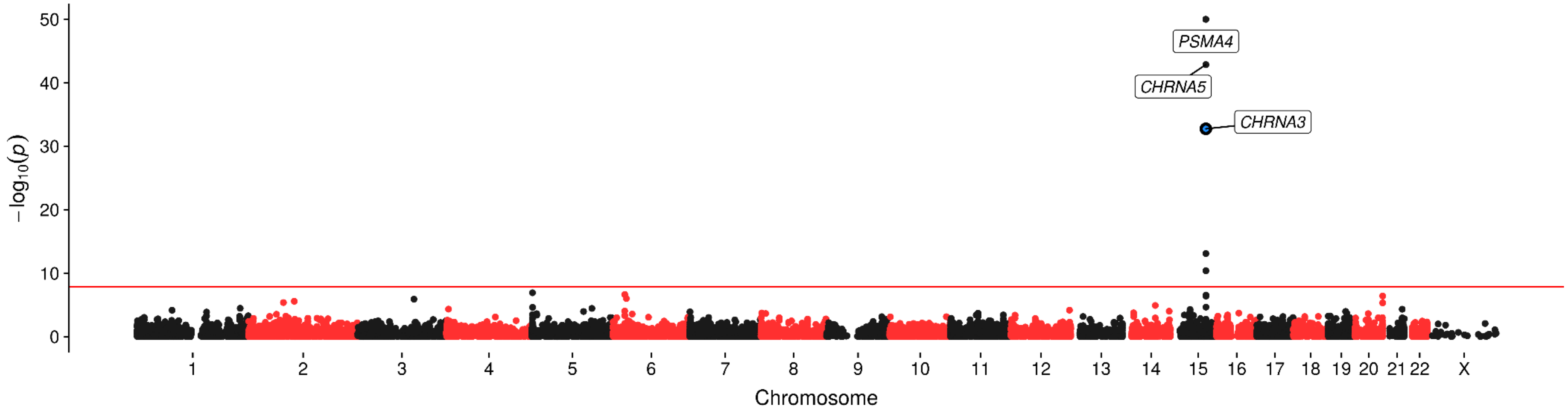

PSMA4
CHRNA5
CHRNA3
−log10(p)
50
40
30
20
10
0
1
2
3
4
5
6
7
8
9
10
11
12
13
14
15
16
17
18
19
20
21
22
X
Chromosome

Squamous cell lung cancer

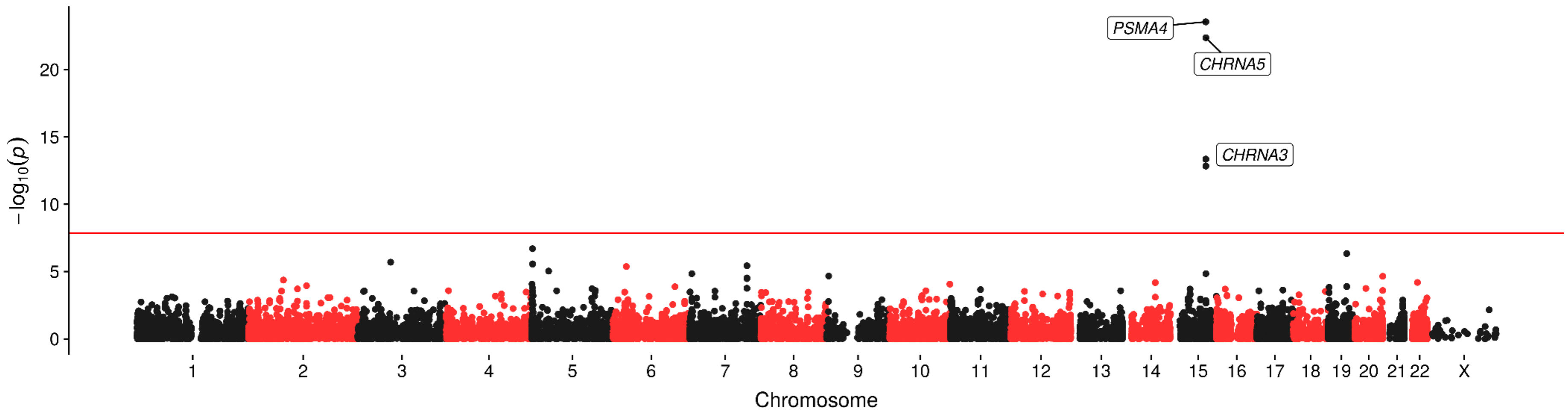

Transferrin

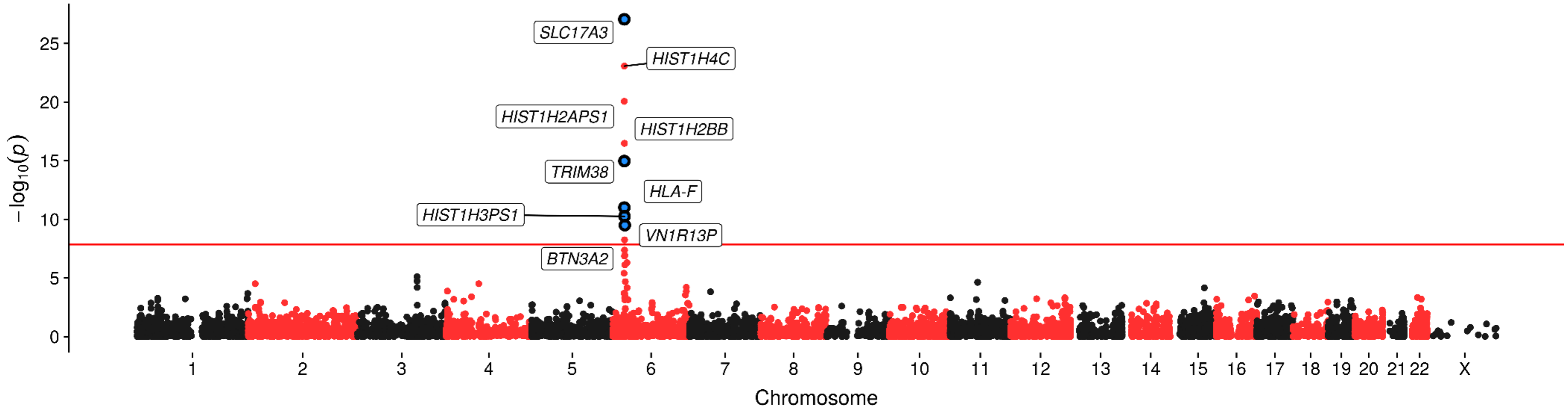

Transferrin Saturation

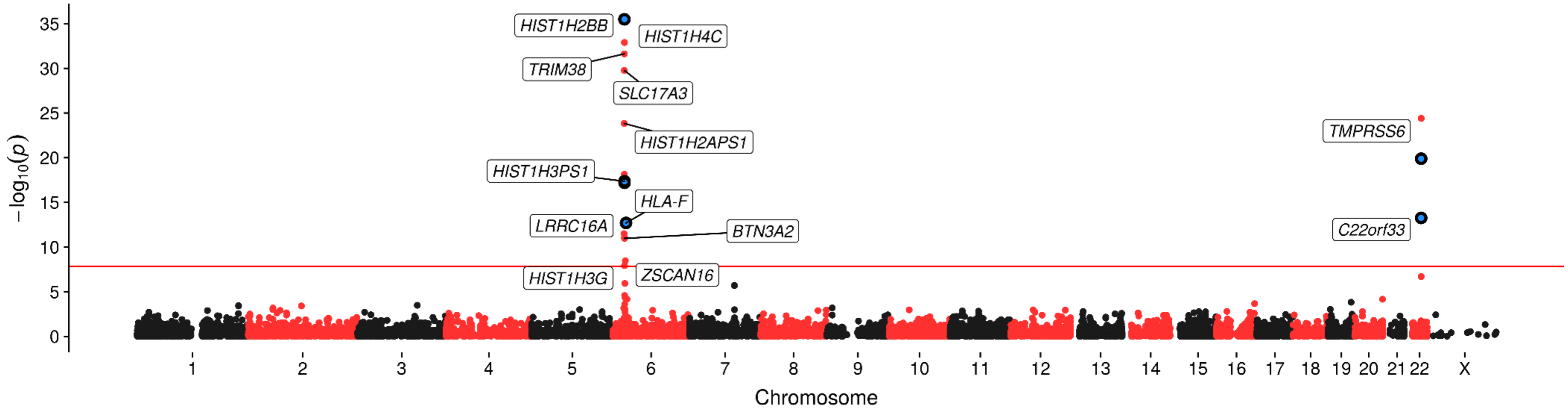

Urate

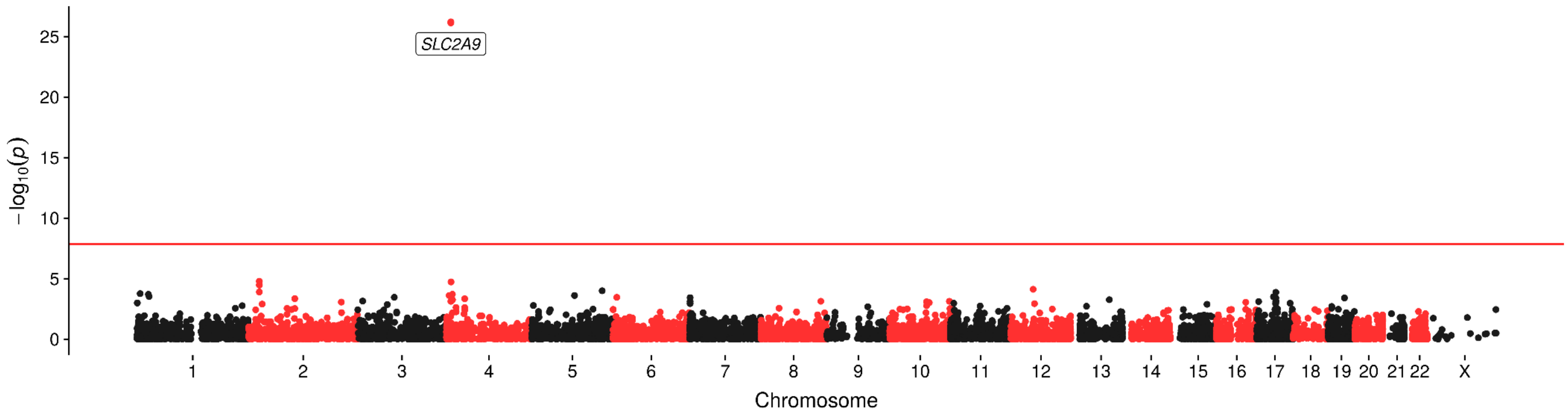

SLC2A9
25
20
15
10
5
0
$-\log_{10}(p)$
1
2
3
4
5
6
7
8
9
10
11
12
13
14
15
16
17
18
19
20
21
22
X
Chromosome
