# Supplementary figures and images for: Systematic Mendelian randomization framework elucidates hundreds of CpG sites which may mediate the influence of genetic variants on disease
Source: Hum Mol Genet. 2018 Jun 8;27(18):3293–304. doi: 10.1093/hmg/ddy210 (PMC6121186; doi:10.1093/hmg/ddy210)

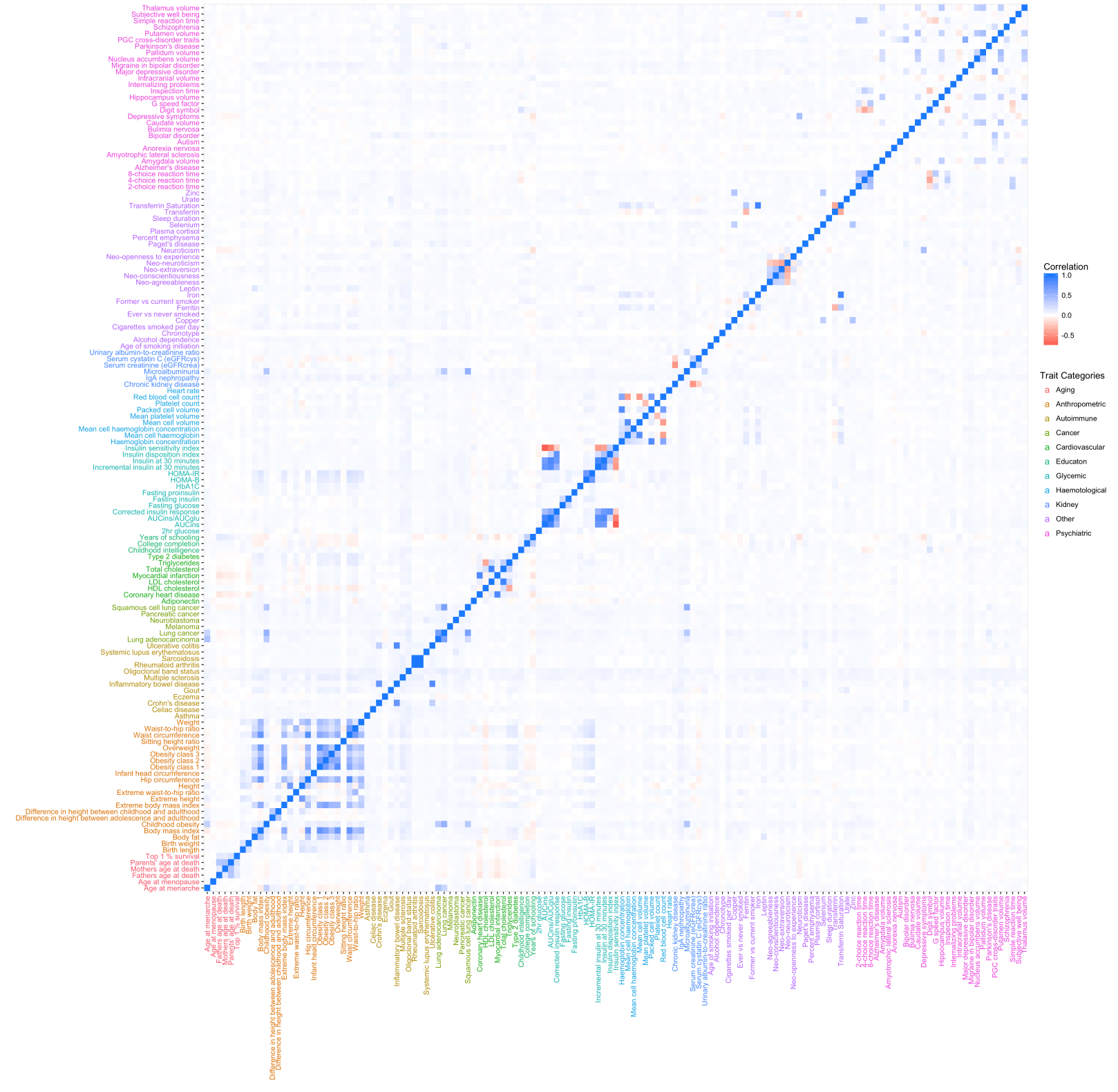

Supplement: Supplementary Data [file ddy210_supp_data.zip › S1 Figure.pdf]

## H3K4me1

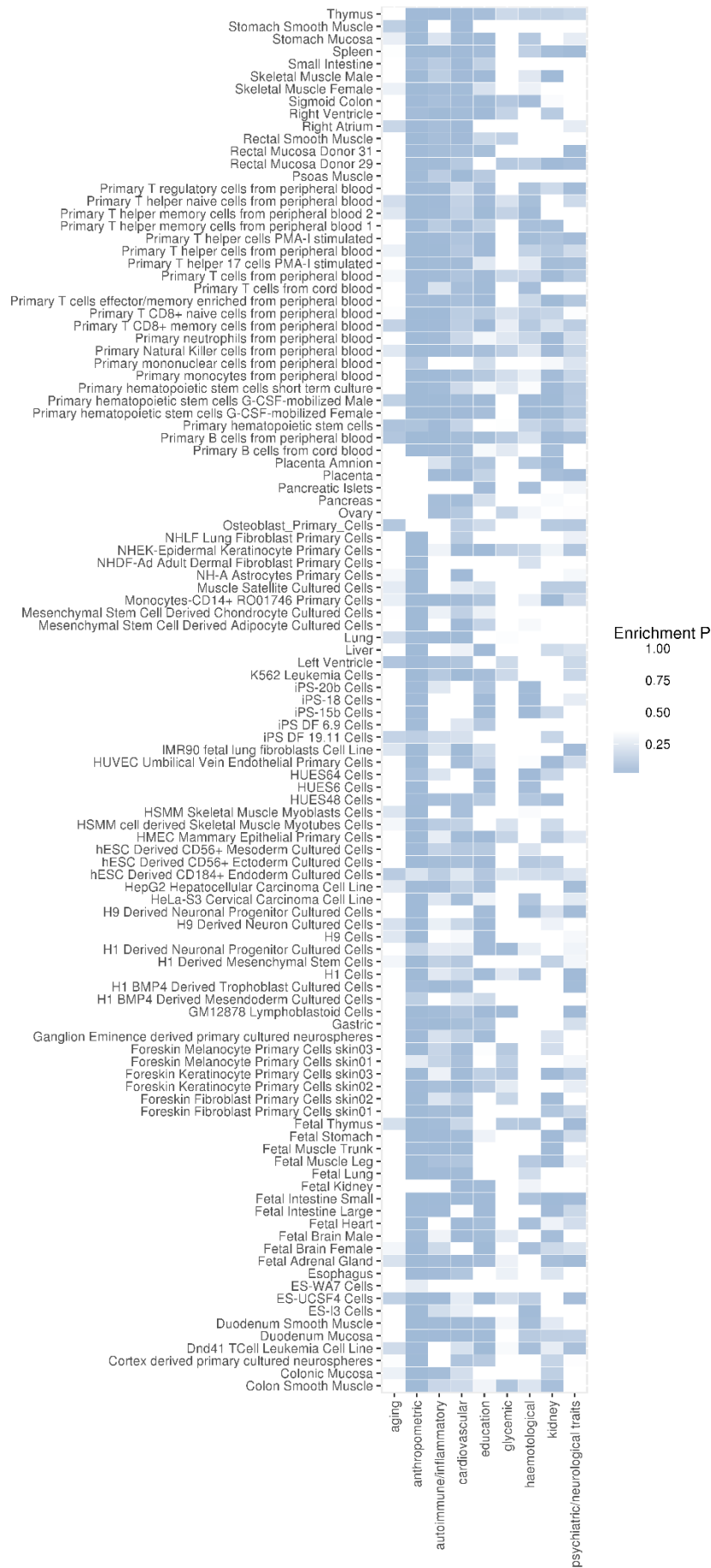

## H3K4me3

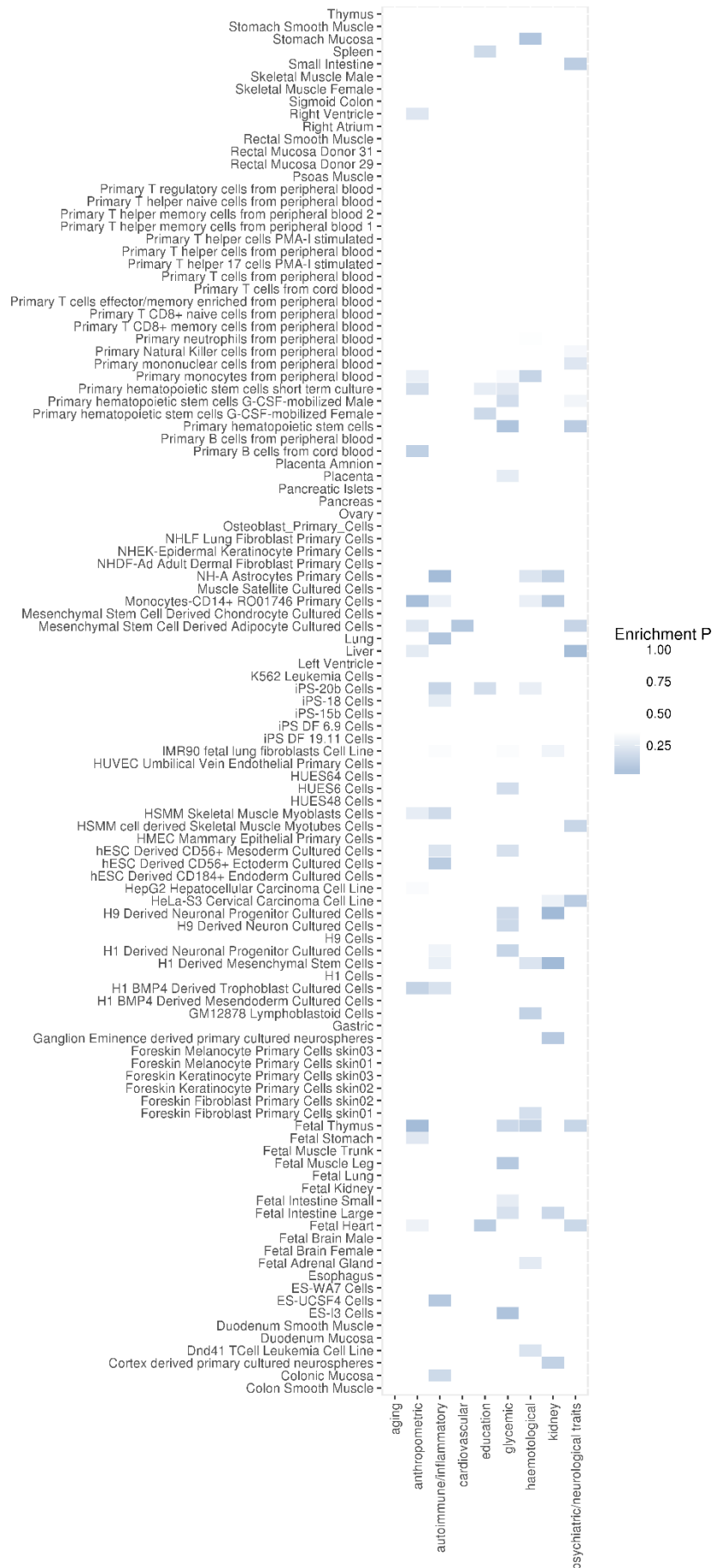

H3K9ac

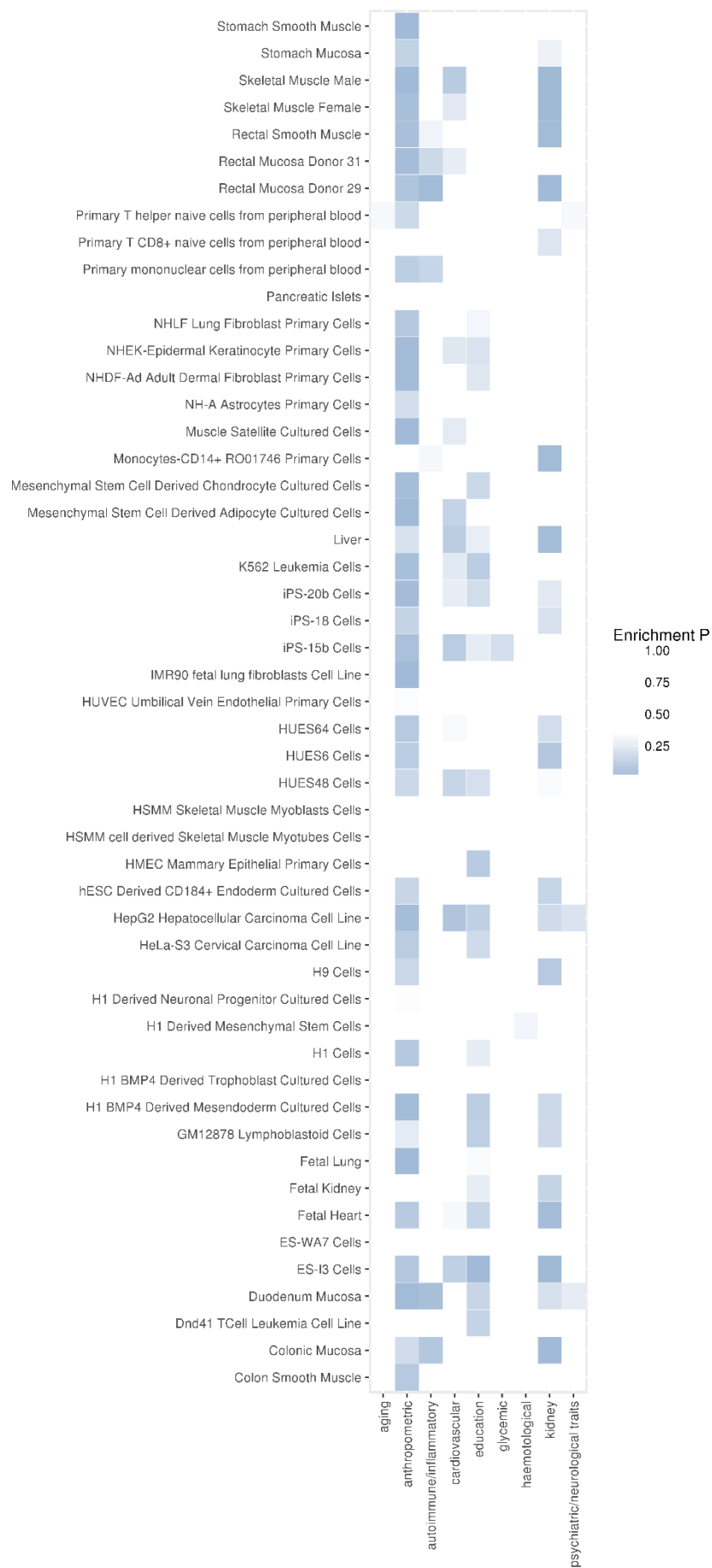

## H3K9me3

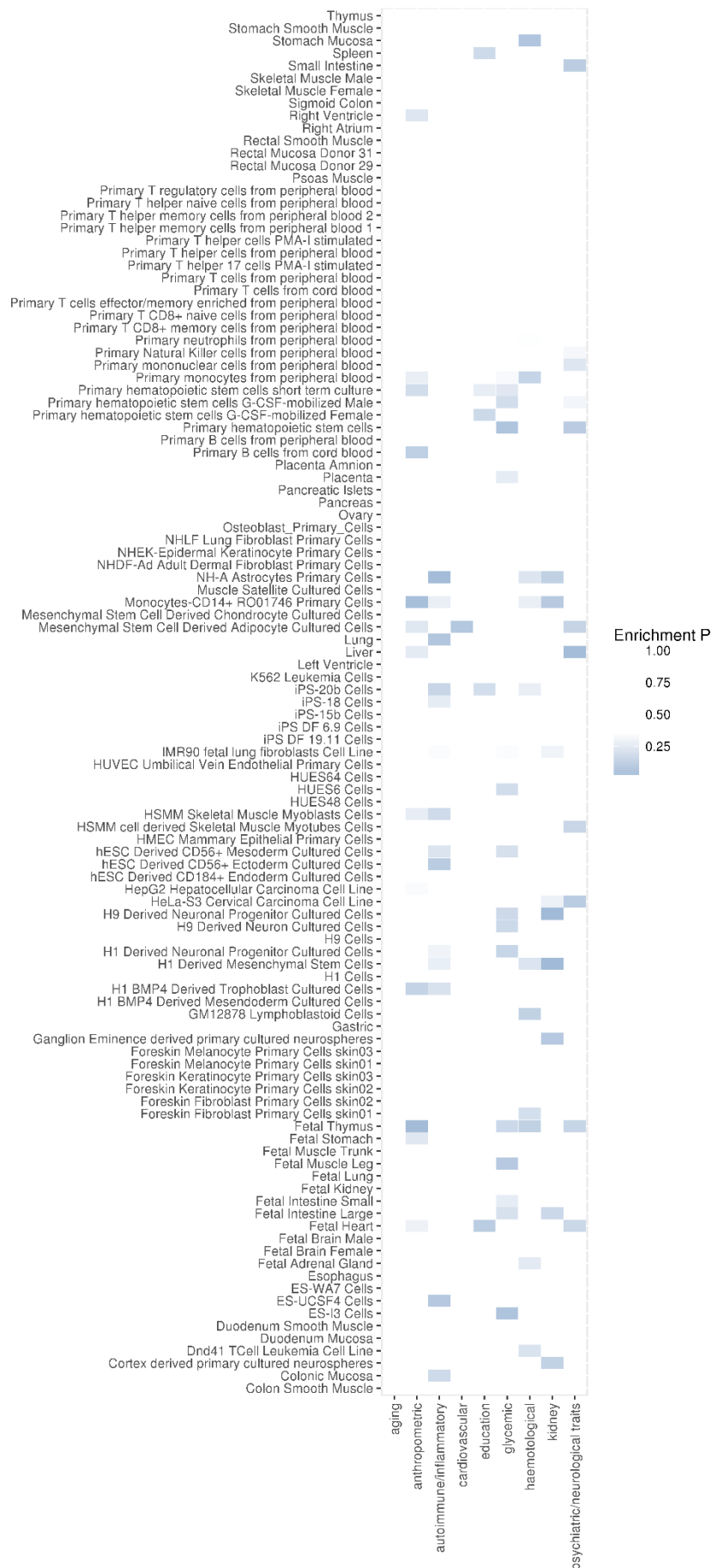

## H3K27ac

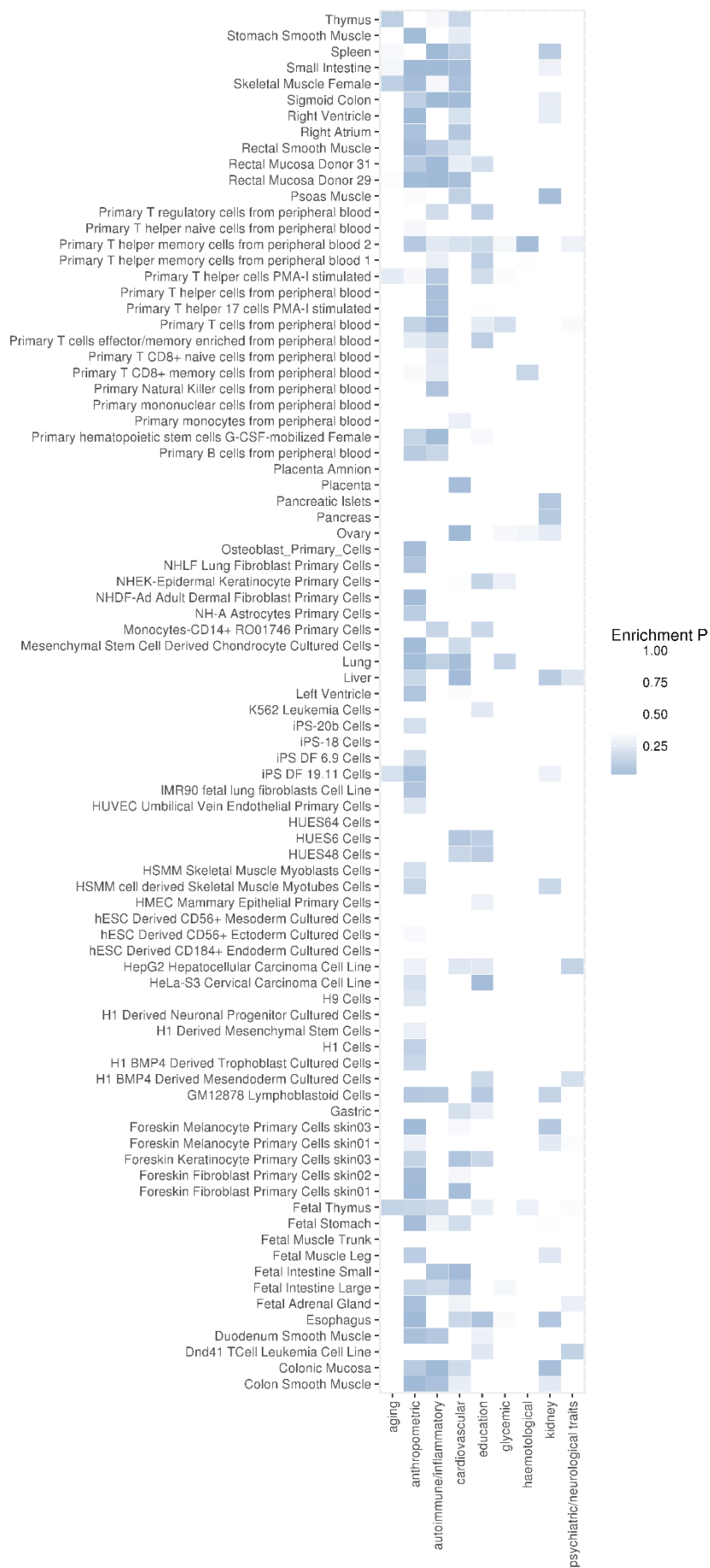

## H3K27me3

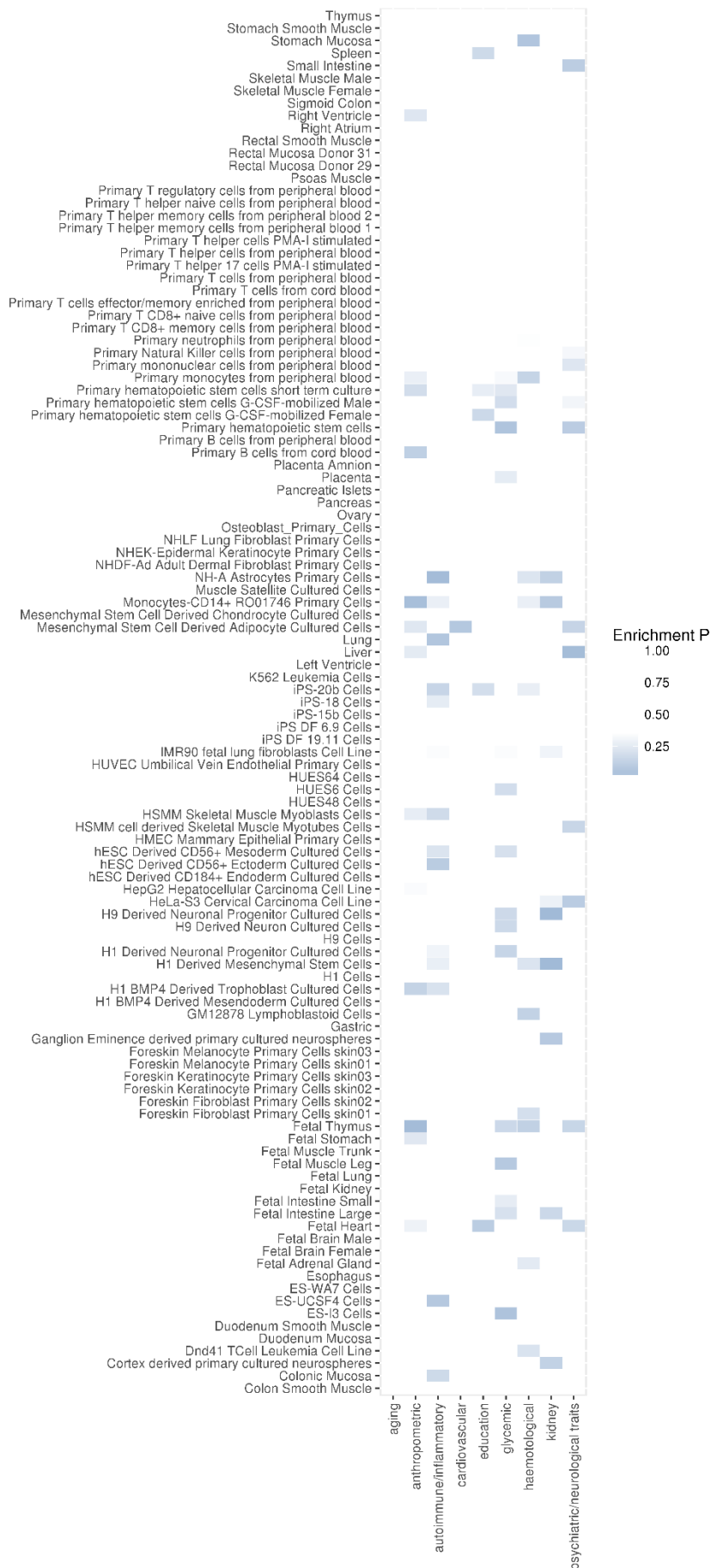

## H3K36me3

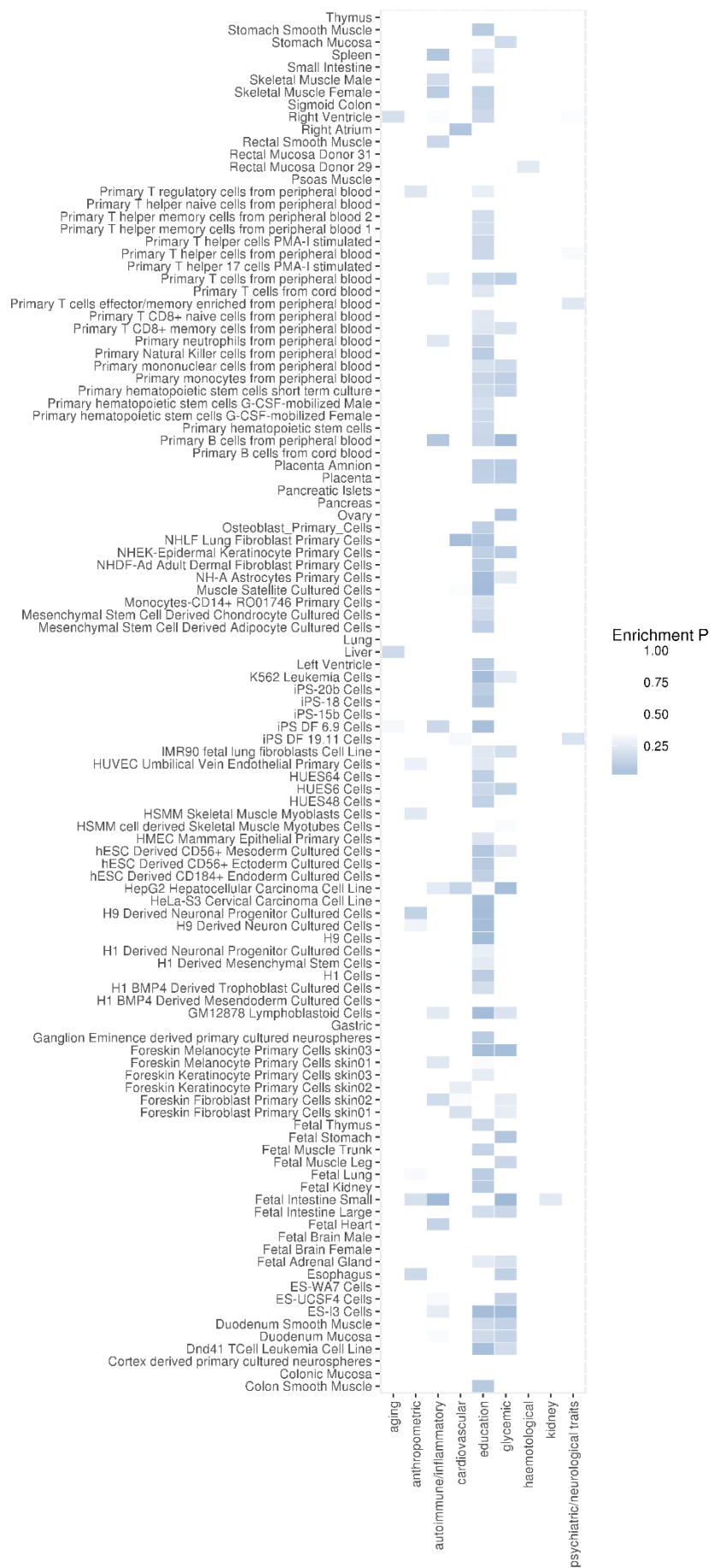

Supplement: Supplementary Data [file ddy210_supp_data.zip › S2 Figure.pdf]
